# Manufacturing Silk Fibroin Hollow Nanoyarns as Fundamental Units for Advanced Medical Textiles

**DOI:** 10.1021/acsami.5c23471

**Published:** 2026-03-17

**Authors:** Athanasios Papakonstantinou, Maria Gabriella Fois, Sergio Acosta, Stephan Rütten, Alexander Kopp, Stefan Jockenhoevel, Alicia Fernández-Colino

**Affiliations:** † Department of Biohybrid & Medical Textiles (BioTex), AME—Institute of Applied Medical Engineering, Helmholtz Institute, 9165 RWTH Aachen University, Aachen 52074, Germany; ‡ Electron Microscopy Facility, Uniklinik RWTH Aachen University, Aachen 52074, Germany; § Fibrothelium GmbH, Aachen 52068, Germany

**Keywords:** silk fibroin, electrospinning, textile, nanoyarn, macrophages, tissue engineering

## Abstract

Nature-inspired designs aim to replicate the hierarchical structure observed in native tissues. Fibers serve as fundamental modular units, enabling the fabrication of complex architectures for the engineering of medical textiles and tissue equivalents. However, synthetic yarns lack inherent biological cues to support tissue integration, leading to a growing interest in yarns derived from natural materials. Here, we describe for the first time the fabrication of hollow nanoyarns from pure silk fibroin (SF) using an advanced funnel electrospinning process. This yielded long SF nanoyarns, spanning several meters, with adequate tensile strength (1.47 MPa) and stretching performance (166.4%). Moreover, the yarns were compatible with autoclaving, permitting effective sterilization and long-term storage, making them suitable for biomedical applications. Indirect cytocompatibility assessment of the scaffolds in accordance with ISO 10993-5 guidelines revealed high metabolic activity for human umbilical vein endothelial cells and human smooth muscle cells, confirming that the scaffolds were nontoxic. Analysis of TNF-α secretion by macrophages showed that the SF scaffolds exhibited low immunogenicity. Furthermore, the structural resilience and flexibility of the yarns supported bottom-up assembly into textile constructs by weaving. This study not only shows for the first time the feasibility of producing SF nanoyarns but also highlights their compelling potential in the field of sustainable and medical textiles.

## Graphical abstract

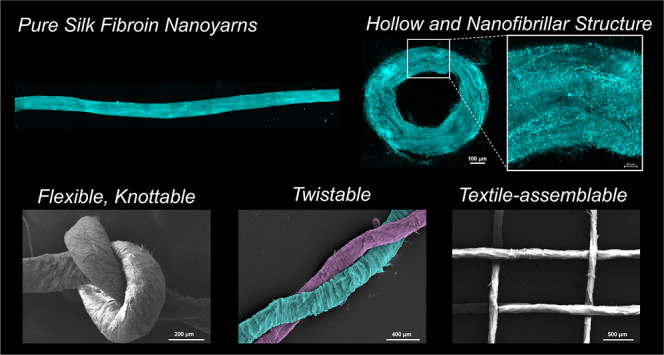

## 1 Introduction

Nature’s approach to construction begins at the most fundamental level, transforming simple chemical components into highly complex and functional structures.^1,2^ Native tissues exemplify this bottom-up strategy, following the same principles of assembly and organization.^3,4^ The extracellular matrix (ECM) is a three-dimensional (3D) and well-structured framework of macromolecules that generally consists of proteoglycans, glycosaminoglycans, and fibrous proteins such as elastin, collagen and fibronectin.^5,6^ The ECM provides mechanical integrity and stability to tissues and organs, enabling them to accommodate physical stress. Beyond this mechanical role, the ECM is also necessary for tissue remodeling and homeostasis, facilitating a range of cellular activities, such as proliferation, migration and differentiation.^7,8^ The multiscale organization of ECM fibrous proteins, beginning at the molecular level (nanoscale) and evolving at the cellular level (microscale) and macroscale into the components of native tissues, underscores the significance of hierarchical structures.^9^ Fibers play a key role in this hierarchy, making them elemental building blocks for the development of tissue equivalents.

Among various fiber-based techniques for the fabrication of scaffolds, electrospinning has gained the attention of researchers because the resulting nanofibrous structures closely resemble the native architecture of the ECM.^8,10,11^ This method produces fibrillar scaffolds with high surface area-to-volume ratios, tunable porosity, and customizable mechanical properties, which are valuable for biomedical applications.^12,13^ The conventional electrospinning setup, which typically produces two-dimensional (2D) scaffolds, has been advanced by incorporating a funnel-shaped collector,^14^ enabling the development of yarn-like structures composed of nanofibers with diameters ranging from a few to several hundred nanometers. The nanoyarns have superior properties compared to traditional textile yarns made from microscale fibers, including a higher specific surface area and aspect ratio, which can enhance cell adhesion. Therefore, nanoyarns are ideal for the assembly of 3D fibrous structures using textile technologies (e.g., braiding, weaving, or knitting), resulting in hierarchical structures across different scales.^15,16^ This is particularly relevant in the fields of medical textiles, tissue engineering, and regenerative medicine. The potential is further enhanced by the ability to produce intricate yarn shapes, such as hollow yarns. Hollow nanostructures with precisely controlled pore volumes and shield thicknesses have an even larger surface area and surface-to-volume ratio than their solid counterparts. In addition, they benefit from a lightweight structure and more flexibility,^17,18^ unlocking new possibilities in biomedicine and drug delivery.

Specifically, in tissue engineering, the tubular geometry of the hollow fibers closely mimics native structures such as the esophagus, intestine, trachea, and blood vessels.^19^ This geometric similarity makes them promising candidates for fabricating artificial tubular tissues and vascular models. In this context, hollow fibers are highly suitable for integration into microfluidic systems. Their dimensions align with small vessels such as arterioles and capillaries, potentially enabling the formation of perfusable vascular networks in organoids.^20^ In this regard, hollow yarns with thin, porous walls are particularly attractive, as these structural features facilitate efficient diffusion of nutrients and oxygen.^21^

The hollow architecture also supports multifunctional design: the sheath can incorporate bioactive or structural components, while the inner core offers space for encapsulation and controlled release of therapeutic agents.^19^ The nanofibrillar shell potentially enables fine control over release kinetics, providing a high surface-area-to-volume ratio to enhance drug-loading efficiency and protect payloads from degradation.

Beyond biomedical applications, hollow fibers also show potential in areas such as thermal insulation and fluid transport.^22^ Their large internal voids can trap air and reduce heat transfer by limiting conduction, convection, and radiation. Hollow fibers can significantly enhance thermal resistance and efficiency, as lightweight, sustainable fillers in building materials such as walls or panels. Simultaneously, their low density and large surface area make hollow fibers attractive for applications in membrane technologies, gas separation systems, and breathable textiles.^22^

Despite the potential of both nanoyarns and hollow counterparts, only a few researchers have fabricated them by implementing the modified electrospinning process discussed above. Examples include a novel setup featuring a rotating funnel target, creating continuous, twisted poly­(l-lactide) yarns,^23^ and another using a rotating funnel and two oppositely charged nozzles to produce continuous and highly twisted nanoyarns from synthetic polymers such as poly­(vinylidene fluoride) (PVDF), polycaprolactone (PCL), polyacrylonitrile (PAN), and polystyrene (PS).^24^ Similarly, PAN, polyvinylidene fluoride trifluoroethylene (PVDF TrFE) and PCL nanoyarns have been fabricated using modified electrospinning setups.^25^ These yarns have predominantly been made from synthetic polymers due to their mechanical strength and processability.^26,27^ However, such materials often fail to replicate the biological milieu of the ECM, potentially leading to poor integration or rejection after implantation.^28–30^

Given the constraints that arise from the use of synthetic materials,^28^ there is an increasing demand for advanced yarns derived from natural, biobased resources.^31–34^ Silk as a textile has significantly influenced human civilization and has also been widely used for medical applications, for example as a suture material.^35^ The best-characterized form of silk is derived from the silkworm *Bombyx mori*, and consists of two polypeptide chains, a light chain (*M*_w_ ∼26 kDa) and a heavy chain (*M*_w_ ∼390 kDa) linked by a disulfide bond, and a glycoprotein P25 (*M*_w_ ∼30 kDa), which binds to them via noncovalent hydrophobic interactions.^36–38^ Several silk-based products have received approval from the Food and Drug Administration (FDA), further highlighting their biocompatibility, biodegradability, and translational potential.^35,39^ For example, SERI Surgical scaffold (for plastic and reconstructive surgery) received 510 (k) clearance in 2014.^35^ Furthermore, the processability of silk facilitates its transformation into diverse configurations, including fibers,^40^ films,^41^ sponges,^42^ hydrogels,^43^ 3D printing structures,^44^ and nanoparticles.^45^ Yet, solutions of reconstituted silk fibroin often exhibit poor spinnability, leading to frequent jet breakup or bead formation during electrospinning. To overcome these limitations, SF is often combined with synthetic polymers such as poly­(l-lactic acid) (PLLA), polylactic-*co*-glycolic acid (PLGA), poly­(caprolactone) (PCL), polydioxanone (PDO), and poly­(l-lactide-*co*-ε-caprolactone) (PLCL)^46–51^ for applications requiring high load-bearing capacity. These blends enhance solution viscosity, stabilize the spinning jet, and overall improve the processability of the mixture.

However, while SF breaks into amino acids, synthetic polymers like epoxies or polyesters in silk composites often cannot be fully broken down by the body, leaving nonabsorbable residues that may require removal or cause chronic inflammation. Therefore, pure SF offers superior biocompatibility and bioactivity for medical textile implants, and adding synthetic polymers might introduce trade-offs in performance and safety. Continuous multimeter yarns have also been fabricated from a blend solution of SF and tropoelastin to generate a woven mesh.^52^ Excluding the second component could simplify processing by avoiding the complexities of blending two proteins during electrospinning, potentially reducing production costs and increasing yarn uniformity. So far, the fabrication of pure SF nanoyarns has been largely overlooked.

Here, we describe the first pure SF hollow nanoyarns produced by funnel electrospinning as a novel fabrication technology. The mechanical and biological properties of these nanoyarns were evaluated, as well as their ability to withstand terminal sterilization. The SF nanoyarns were also used to fabricate hierarchically organized structures by weaving, thereby showcasing their potential for the construction of multiscale systems. This approach not only demonstrates the feasibility of manufacturing hollow SF nanoyarns but also underscores their promising role in the development of medical textiles.

## 2 Materials and Methods

### 2.1 Preparation of Spinning Dope

The SF solution was provided as an aqueous solution using PureSilk technology (Fibrothelium, Aachen, Germany). The stock concentration was 16–22.8% (w/v). The solution was diluted to 4% (w/v) with ultrapure water type I. The diluted solution was transferred to 24-well plates and stored at −20 °C overnight, then lyophilized at −20 °C and 0.05 mbar for 48 h using an Alpha 2–4 LSCPlus (Martin Christ Gefriertrocknungsanlagen, Osterode am Harz, Germany) to obtain regenerated SF sponges. These were dissolved in 98% formic acid (Carl Roth, Karlsruhe, Germany) for 3 h to prepare a 15% (w/v) SF/formic acid solution as the spinning dope.

### 2.2 Funnel Electrospinning

Electrospinning was carried out using a custom-made setup (Figure 1a). Briefly, it consisted of two PCSC/XP6HEDMX high-voltage power supplies (Eltex electrostatic, Weil am Rhein, Germany), two LA-100 syringe pumps (Landgraf Laborsysteme HLL, Langenhagen, Germany) where 1 mL plastic syringes (Becton Dickinson, Heidelberg, Germany) were mounted and connected to 21-gauge blunt needles (B. Braun, Hessen, Germany) with the help of a tube connector system, a rotating funnel as a collector with a diameter of 57 mm, and a maximum rotation speed of 1200 rpm. For the take-up process, we used a winder with a diameter of 20 mm and a maximum rotation speed of 12 rpm. A feeder unit containing a commercial poly­(vinyl alcohol) (PVA) multifilament yarn (U.+M. Schmidt, Freiburg, Germany) was placed behind the funnel collector. During the process, a dense network of SF nanofibers was generated in the periphery of the funnel. At the same time, the multifilament PVA was wound by the take-up unit, resulting in a core–sheath structure. The system parameters were optimized for the formation of a stable Taylor cone. The spinnerets were aligned in parallel (0°) with the plane of the funnel collector, and the pump rate was set at 0.5 mL/h, whereas the distance between the funnel collector and the spinnerets was maintained at 10 cm. The applied voltage was adjusted to ±12.5 kV. Additionally, the rotation speed of the funnel collector was maintained at 450 rpm, and the winder at a speed of 4.5 rpm. The nanoyarns were categorized according to the number of iterations of the process (5 or 10 spinning cycles), followed by immersion in absolute ethanol to achieve β-sheet crystallization and insolubilization. Finally, the SF nanoyarns were immersed in ultrapure water at 55 °C to dissolve the PVA core, leaving hollow SF nanoyarns.

**Figure 1:**
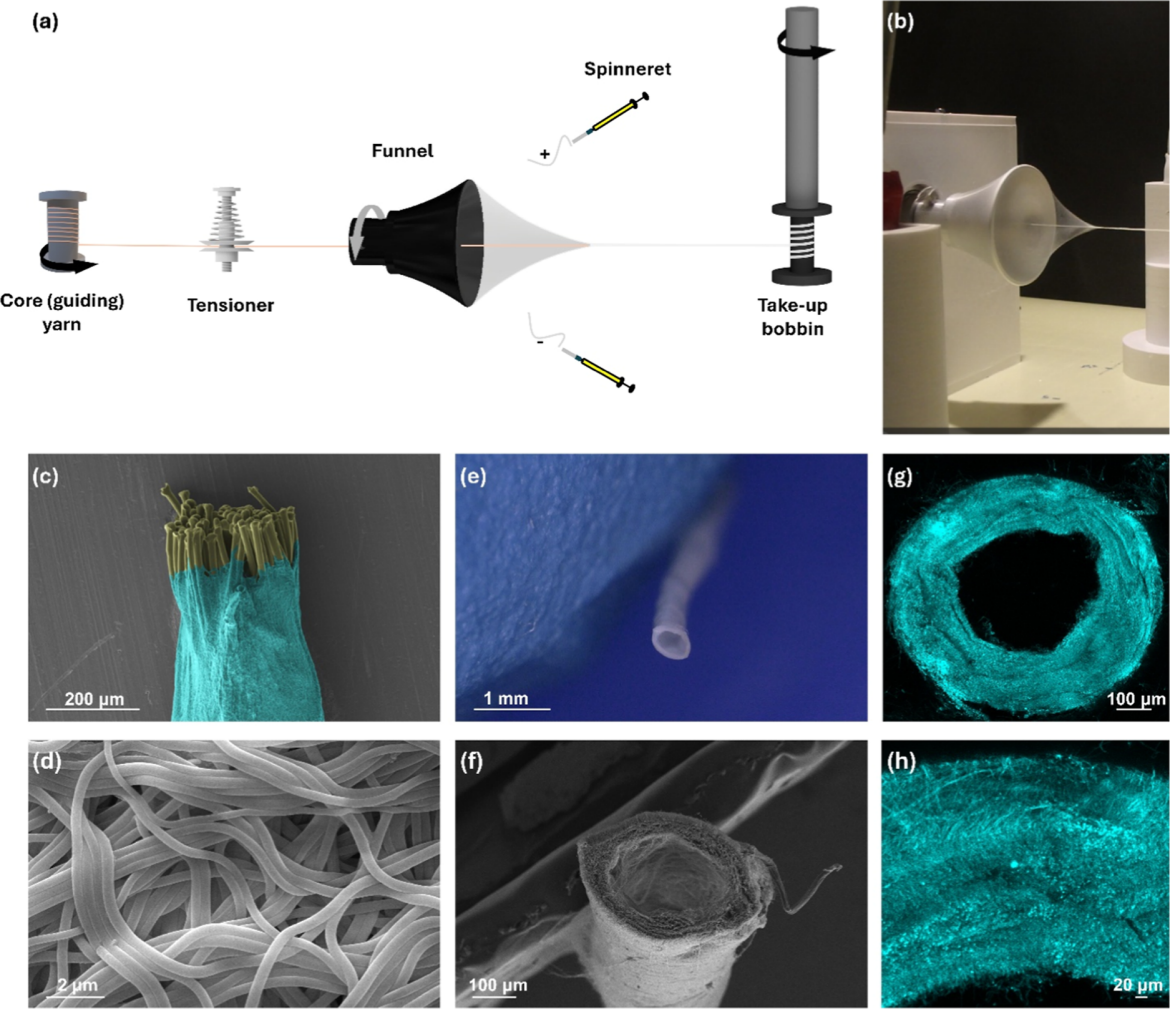
Fabrication of silk fibroin (SF) nanoyarns. (a) Schematic representation of the electrospinning setup for the fabrication of SF nanoyarns. (b) Video frame showing the formation of the nanofiber cone. Funnel diameter is 57 mm. (c) SEM image of the core–sheath structure (inner PVA yarn shown in yellow, and outer sheath in turquoise, using MountainsSEM. (d) Detailed SEM image of the SF nanofiber structure that forms the sheath of the hollow nanoyarn. (e) Optical microscopy cross-section of the hollow SF nanoyarn. (f) SEM cross-section of hollow SF nanoyarns. (g) Confocal image of the SF hollow nanoyarn cross-section. (h) Zoomed-in view of the sheath (cross-section).

### 2.3 Scanning Electron Microscopy (SEM)

Samples were dried at room temperature or by critical point drying and then sputter-coated with a 20 nm gold–palladium layer using an EM SC D500 instrument (Leica Microsystems, Wetzlar, Germany). Images were captured using a Quattro S microscope (Thermo Fischer Scientific, Darmstadt, Germany) with an accelerating voltage of 10 kV. MountainsSEM software (courtesy of Digital Surf, Besançon, France) was used to color the images.

### 2.4 Confocal Microscopy

Images were acquired using an inverted LSM 980 with Airyscan 2 (Zeiss, Oberkochen, Germany) operated in CO–8Y (cross section) or SR-4Y (zoom in view) mode. The system was equipped with an Airyscan 2 detector and a Plan-Apochromat 10×/0.45 numerical aperture (NA) objective (Zeiss). Fibers were visualized based on their intrinsic autofluorescence, which was excited with a 405 nm diode laser, and the emitted fluorescence was collected at 380–735 nm. Z-stack images covering ∼35 μm in depth were acquired at intervals of 1.3 μm. Image data sets were processed using automated 3D Airyscan deconvolution and visualized as maximum intensity projections. All image acquisition, deconvolution, and projection steps were carried out using ZEN Blue v3.9 (Zeiss).

### 2.5 Sterilization of the SF Hollow Nanoyarns

SF nanoyarns were processed by steam sterilization at 121 °C and 200 kPa for 20 min using a Systec DE-23 autoclave (Systec, Linden, Germany).

### 2.6 Morphological Characterization

To calculate the outer diameter, we visualized nanoyarns under a digital microscope (Keyence Deutschland, Neu-Isenburg, Germany). Briefly, five measurements were taken at different points along the nanoyarn, and the average diameter was calculated. To calculate the inner diameter, samples were fixed with Carnoy’s solution, embedded in paraffin, and cut into 3 μm sections. Images were acquired using an AxioCam MRc digital camera and brightfield microscope (Zeiss). The images were analyzed using ImageJ software.^53^

### 2.7 Swelling Ratio

To assess the swelling properties of the SF nanoyarns, the nanoyarns were cut into segments of 1.1 cm in length, cross-linked with ethanol, and subsequently autoclaved. The samples were then lyophilized for 6 h to remove the water, and the dry weight (*W*_d_) was measured. For wet (*W*_w_) weights, the samples were immersed in ultrapure water Type I at room temperature for 30 min. All excess water was removed using KIMTECH SCIENCE laboratory wipes (KIMBERLY-CLARK GmbH, Koblenz, Germany) from the surface and the inner lumen. For the measurements, an analytical balance, Mettler Toledo New Classic MF (Mettler-Toledo GmbH, Greifensee, Switzerland), was used. The swelling ratio was calculated using eq 1

1 $$SR\;\left(\%\right)=\frac{W_{w}-W_{d}}{W_{d}}\times 100$$

### 2.8 Mechanical Characterization

For mechanical characterization, we used a Univert tensile test device (CellScale Biomaterials Testing, Waterloo, Canada), equipped with a 1 N load cell, with the clamps at a distance of 2–4 cm. All measurements were conducted in a bath of Dulbecco’s phosphate buffered saline (DPBS; Thermo Fisher Scientific, Darmstadt, Germany) heated to 37 °C. The nanoyarns were immersed in DPBS overnight, then uniaxially stretched at a constant strain rate of 0.156 mm/s until failure. The stress–strain curve was plotted, and ultimate tensile strength (UTS), elongation to break, and Young’s modulus were calculated (*n* = 5 samples per condition).

### 2.9 Fourier Transform Infrared (FTIR) Spectroscopy

Conformational changes in SF nanoyarns were investigated by FTIR spectroscopy using a Spectrum 3 FTIR spectrometer (PerkinElmer, Waltham, MA, USA). Absorbance was measured at 400–4000 cm^–1^, with 64 scans. The deconvolution of the infrared spectra targeted the amide I (1600–1700 cm^–1^) region using Gaussian peaks. Specifically, a linear baseline was first defined and subtracted. The second derivative of the spectrum was calculated to identify the initial number and positions of subpeaks. These peak positions (4 peaks per sample) were used as initial constraints for nonlinear curve fitting using a Gaussian model and the Levenberg–Marquardt algorithm. The fitting was iterated until a satisfactory coefficient of determination (*R* ^2^ > 0.99) was achieved. Β-sheet content was calculated as the ratio of the integrated area of the β-sheet-related peaks (1610–1627 cm^–1^ and 1696–1700 cm^–1^) to the total integrated area of the amide I band.

### 2.10 SF Nanoyarns Degradation in Aqueous Environment

Electrospun SF nanoyarns were cut into 1 cm lengths, cross-linked with ethanol, and sterilized by autoclaving before in vitro degradation testing. The samples were immersed in 0.5 mL HyPure Cell Culture (Sterile) grade water (HyClone Laboratories, South Logan, Utah, USA) and incubated at 37 °C for 2 weeks. To quantify SF degradation, the concentration of released protein in the incubation water was determined by measuring absorbance at 205 nm (corresponding to peptide bonds) using a NanoDrop One spectrophotometer (Thermo Fisher Scientific). A standard curve was generated using known concentrations of SF. After the nanoyarns were dried and weighed, the amount of SF released into the incubation water was calculated from the standard curve and expressed as a percentage of the final dry mass of the nanoyarns.

### 2.11 Cell Isolation and Culture of HUVECs and HUASMCs

Human umbilical vein endothelial cells (HUVECs) were isolated from human umbilical cords as previously described.^54^ The umbilical cords were provided by the RWTH Aachen University Centralized Biomaterial Bank (cBMB), in compliance with its regulations, following the RWTH Aachen University Medical Faculty Ethics Committee approval (cBMB project number 323). The HUVECs were seeded in flasks coated with 2% (v/v) gelatin (Merck, Darmstadt, Germany) and cultured in endothelial growth medium 2 (EGM-2) supplemented with fetal calf serum (FCS), epidermal growth factor, basic fibroblast growth factor, insulin-like growth factor, vascular endothelial growth factor 165, ascorbic acid, heparin, and hydrocortisone (supplied ready-to-use by PromoCell, Heidelberg, Germany). The HUVECs were cultured at 37 °C in a humidified 5% CO_2_ atmosphere. Once the cells reached ∼80% confluence, they were trypsinized using 0.05% trypsin/0.02% ethylenediaminetetraacetic acid (EDTA) and frozen in liquid nitrogen.

Human umbilical artery smooth muscle cells (HUASMCs) were obtained from umbilical cords as previously described,^55^ and under the ethical approval procedure stated above. Briefly, the artery was washed with DPBS, and the endothelial cells were removed using 1 mg/mL collagenase. The adventitia was then removed, and the artery was minced into 1 mm rings and bathed in Dulbecco’s modified Eagle’s medium (DMEM; Thermo Fisher Scientific) supplemented with 10% FCS (Capricorn Scientific, Ebsdorfergrund, Germany; lot no. CP21-4355). The cells were serially passed using trypsin/EDTA as above and cultured at 37 °C and 95% humidity in a 5% CO_2_ atmosphere. The cells were frozen at passage 3 in liquid nitrogen.

### 2.12 Cytocompatibility Assay

Cell viability was evaluated according to ISO-10993-5,^56^ with an indirect test. Scaffold samples (15% SF electrospun mats treated with ethanol) were washed five times with DPBS under sterile conditions to remove ethanol residues. Then, they were immersed in cell media (6 cm^2^/mL) as stated in ISO10993-12,^57^ using 12-well cell culture plates (Avantor, Schwerte, Germany). At the same time, HUVECs were thawed and cultured as described above in 96-well cell culture plates (Avantor) at a seeding density of 6 × 10^3^ cells/well and cultured for 1 day in EGM-2 supplemented with 1% (v/v) Anti–Anti (100X) streptomycin/penicillin (Thermo Fisher Scientific). In parallel, HUASMCs were seeded into 96-well cell culture plates at a density of 3 × 10^3^ cells/well and cultured for 1 day in DMEM containing 10% (v/v) FCS and 1% (v/v) streptomycin/penicillin at 37 °C. After 1 and 3 days of scaffold-medium incubation, extracts were collected, vortexed for 15 s, and fed to the cells. The cells were then incubated for 1 and 3 days as above. Latex extracts (6 cm^2^/mL) and 50% dimethyl sulfoxide (DMSO) in medium were used as cytotoxic-positive controls, also after 1 and 3 days, whereas cell culture medium alone (EGM-2 or DMEM) was used as the cytotoxic-negative control.

At each time point, the metabolic activity of both cell types was measured using a CellTiter-Blue Cell Viability Assay (Promega, Madison, WI, USA) according to the manufacturer’s instructions. Following the addition of 20 μL Cell-Titer Blue reagent to each well containing cells precultured with the extracted media, the cells were incubated at 37 °C for 4 h in the dark, and fluorescence (560Ex/590Em) was measured using a Tecan plate reader (Tecan Deutschland, Crailsheim, Germany). The fluorescence values of HUVECs and HUASMCs exposed to cell culture medium alone were considered as 100% viable, and all other results were normalized accordingly.

To assess cell morphology, HUVECs were washed with DPBS and fixed with 10% formaldehyde (Carl Roth) for 15 min at room temperature. The cells were then permeabilized with 0.1% (v/v) Triton X-100 (Sigma-Aldrich, Steinheim, Germany) in DPBS for 30 min at room temperature. Nonspecific binding sites were blocked with 1% (w/v) bovine serum albumin (BSA; Sigma-Aldrich) in DPBS for 1 h at room temperature. Next, the HUVECs were incubated for 1 day at 4 °C with a monoclonal mouse anti-CD31 primary antibody (Sigma-Aldrich) in 0.1% (w/v) BSA in DPBS diluted 1:500. The next day, cells were thoroughly washed in DPBS and incubated overnight at 4 °C with the goat antimouse Alexa Fluor 568-conjugated secondary antibody (Abcam, Cambridge, UK) in 0.1% (w/v) BSA diluted 1:500, together with 4′,6-diamidino-2-phenylindole (DAPI; Carl Roth) and phalloidin-iFluor 488 (phalloidin; Cayman Chemicals, Ann Arbor, MI, USA) at dilutions of 1:2500 and 1:500, respectively. The stained cells were visualized using an Axio Observer fluorescence microscope (Zeiss).

The HUASMCs were washed with DPBS and fixed with 10% formaldehyde (Carl Roth) for 15 min at room temperature. The cells were then permeabilized with 0.1% (v/v) Triton X-100 in DPBS for 30 min at room temperature. The actin cytoskeleton and cell nuclei were stained overnight at 4 °C with phalloidin and DAPI at dilutions of 1:500 and 1:2500, respectively. The stained cells were visualized using an Eclipse Ti fluorescence microscope (Nikon Instruments, Tokyo, Japan).

### 2.13 Interaction of Macrophage-Like Cells (U937) with SF Scaffolds

U937 cells were cultured in RPMI 1640 medium (Thermo Fisher Scientific), supplemented with 1 mM sodium pyruvate (Sigma-Aldrich), 10% (v/v) FBS (Capricorn), and 1% (v/v) Anti–Anti (100X) streptomycin/penicillin (Thermo Fisher Scientific), at 37 °C and 5% CO_2_. The day before the experiment, SF nanoyarns and mats (with a diameter of 6 mm) were thoroughly washed 5 times in DBPS and autoclaved (121 °C, 200 kPa). On the experiment day, SF nanoyarns and mats were placed at the bottoms of low-binding 48-well plates (Greiner Bio-One GmbH, Frickenhausen, Germany). At the same time, fibrin gels (5 mg/mL) were formed, consisting of 1000 μL fibrinogen solution (10 mg/mL in Tris-buffered saline (TBS)), polymerized by 1000 μL thrombin solution (150 μL thrombin 40 U/mL, 150 μL calcium chloride (CaCl_2_), and 700 μL TBS). Fibrinogen was purchased from CSL Behring GmbH (Marburg, Germany); thrombin, TBS, and CaCl_2_ from Sigma-Aldrich. U937 cells (passage 11) were seeded on the scaffolds at a seeding density of 5 × 10^5^ cells/cm^2^ and were differentiated into adherent macrophage-like cells by the addition of 100 nM PMA for 1 and 3 days of culture. Cells seeded on tissue culture plastic (TCP) served as a control. On TCP, macrophage polarization to either the pro-inflammatory (M1) or anti-inflammatory (M2) subtype was obtained by adding lipopolysaccharide (LPS; 100 ng/mL) and interferon (IFN)-γ (20 ng/mL) or interleukin (IL)-4 and IL-13 (both 20 ng/mL) to the PMA-supplemented medium. LPS was purchased from Sigma-Aldrich, and IFN-γ, IL-4, and IL-13 from Peprotech (Thermo Fisher Scientific, Cranbury, NJ, USA).

After U937 cell culture, cell supernatants (*n* = 5) were collected after 1 and 3 days of culture and screened for the release of tumor necrosis factor α (TNF-α) via enzyme-linked immunosorbent assay (ELISA; R&D Systems, Minneapolis, MN, USA), according to the manufacturer’s instructions. Results are presented as concentration in picograms per milliliter.

Macrophage adhesion onto the SF electrospun mat was evaluated after 1 and 3 days of culture through immunofluorescence staining. Briefly, cells were fixated in warmed 4% formaldehyde for 15 min and permeabilized using 0.1% (v/v) Triton X-100 for 30 min at room temperature. To avoid nonspecific binding, cells were blocked with 1% (w/v) BSA for 1 h at room temperature. Next, cells were incubated with Draq5 (1:1000; Life Technologies, Carlsbad, CA, USA) and phalloidin (1:1000; Cayman Chemicals) overnight at 4 °C. SF nanoyarns and mats were imaged using 35 mm μ-dishes (ibidi GmbH, Gräfelfing, Germany) with a drop of Dako fluorescence mounting medium (Agilent Technologies INC., Santa Clara, CA, USA). Cell imaging was performed by confocal laser scanning fluorescence microscopy (LSM 710, Zeiss). For all samples, a Z-stack with 2 μm-thick slices was acquired.

### 2.14 Determination of Endotoxin Levels of SF Nanoyarns

The concentration of endotoxin in the SF nanoyarns was measured using the Pierce Chromogenic Endotoxin Quant kit (Thermo Fisher Scientific) according to the manufacturer’s instructions. Briefly, the samples were washed twice in endotoxin-free water and immersed in 1 mL endotoxin-free water at room temperature for 1 h. The endotoxin standard solution was reconstituted at 10 EU/mL and subsequently diluted to obtain a 1 EU/mL stock solution. This stock solution was used to prepare a series of endotoxin solutions with endotoxin concentrations of 0.5, 0.25, and 0.1 EU/mL. The Amebocyte Lysate solution was reconstituted with 1.7 mL of endotoxin-free water. As a positive control, 1 ng/mL of LPS was used. As a negative control, 1 mL of endotoxin-free water was used. After 1 h, 50 μL of each endotoxin standard solution, positive and negative control, and samples were added to a prewarmed 96-well plate at 37 ± 1 °C. Subsequently, 50 μL of the Amebocyte Lysate solution was added to each well, mixed gently, and incubated for 14 min at 37 ± 1 °C. The Chromogenic Substrate was reconstituted with 3.4 mL of endotoxin-free water and mixed gently. After the 14 min of incubation, 100 μL of the Chromogenic Substrate was added to each plate, mixed gently, and followed by incubation at 37 ± 1 °C for 6 min. The reaction was stopped by adding 50 μL of 25% of acetic acid (Sigma-Aldrich, Steinheim, Germany) to each well. The plate was mixed gently and transferred immediately to a Tecan Infinite M200 reader (Tecan Deutschland GmbH, Crailsheim, Germany). Optical density was measured at 405 nm.

### 2.15 Feasibility of the Bottom-Up Approach

SF nanoyarns were manually assembled into knots and woven meshes to evaluate flexibility and structural integrity. For knot fabrication, nanoyarns were tied consistently using a simple knot configuration. For mesh fabrication, nanoyarns were interlaced manually using tweezers, in a custom-made frame consisting of pins, as previously explained.^52^ Scanning electron microscopy and brightfield microscopy were used to visualize the constructs in dry conditions.

### 2.16 Statistical Analysis

All experiments featured at least *n* = 3 replicates. Data are presented as means ± standard deviations (SD). Statistical analysis was carried out using GraphPad Prism 8 (GraphPad Software, Boston, MA, USA) with Welch’s *t*-test or one-way analysis of variance (ANOVA) followed by Tukey’s post hoc test for multiple comparisons. In all figures, significance is indicated as follows: *p < 0.05, **p < 0.01, ***p < 0.001, and ****p < 0.0001, whereas *p*-values ≥ 0.05 are defined as nonsignificant (ns).

## 3 Results

### 3.1 Fabrication of SF Hollow Nanoyarns

We devised a custom-made electrospinning setup that enables a guiding PVA yarn to be threaded through a pinhole along the cone’s axis (Figure 1a). The guiding yarn was retrieved at a constant rate from a feeder (bobbin with core yarn and tensioner) and was collected from a take-up bobbin, while electrospun SF nanofibers were wrapping around it. The nanofibers were deposited initially onto the rim of the rotating funnel and evolved into a conical bundle of fibers due to a combination of electrostatic forces and mechanical motion (Figure 1a,b). The threading of the guiding yarn allowed the SF nanofibers to wrap around it. The tip of this conical nanofiber network was continuously drawn away by the winder, assisted by the continuous retrieval of the guiding yarn and additional tension. This resulted in the constant production of nanofiber yarns. Therefore, as additional SF nanofibers were deposited, they were incorporated into the elongating nanoyarn.

Funnel electrospinning requires the adjustment of a broader set of variables compared to conventional solvent electrospinning. In addition to optimizing standard parameters such as polymer concentration, flow rate, applied voltage, and the distance between the tip and collector, it is also necessary to fine-tune factors unique to the funnel setup. These include the orientation and spacing of the jets relative to the cone, the take-up speed, and the cone’s dimensions and rotation speed. SF nanofibers were generated from oppositely charged spinnerets and deposited in the center and periphery of the funnel-shaped collector. The fabrication of the electrospun yarns was enabled when the flow rates from both spinnerets were 0.5 mL/h, the voltage was 12.5 kV, the distance between the funnel collector and spinnerets was 7.5 cm, and the angle of the spinnerets was 0°. The funnel and winder rotated at 450 and 4.5 rpm, respectively. Both the formation of a stable fibrous cone on the funnel and the stability of the guiding yarn are important to maintain an uninterrupted process. A tensiometer was therefore placed behind the funnel to ease the retrieval of the PVA yarn.

The resulting yarns therefore comprised a PVA core and an outer sheath of SF nanofibers (Figure 1c). Immersion in ethanol for 2, 6, and 24 h induced the structural stabilization of SF through solvent-mediated self-assembly into β-sheet-rich domains,^58^ as confirmed by FTIR analysis (Figure 2). This conformational transition served as a physically cross-linking mechanism, rendering the SF sheath water insoluble. Subsequent immersion in ultrapure water (55 °C) dissolved the PVA core to form a hollow SF electrospun nanoyarn (Figure 1e–h). Repeating the electrospinning process for 5 or 10 spinning cycles produced yarns with varying outer diameters (Figure S1 and Table S1), reflecting differences in the thickness of the sheath. The sheath was composed of nanoscale fibers (231.44 ± 31.89 nm in diameter). As expected, the diameter of the inner core of the nanoyarn remained constant (342.7 ± 53.4 μm), matching the dimensions of the initial PVA guide yarn.

**Figure 2:**
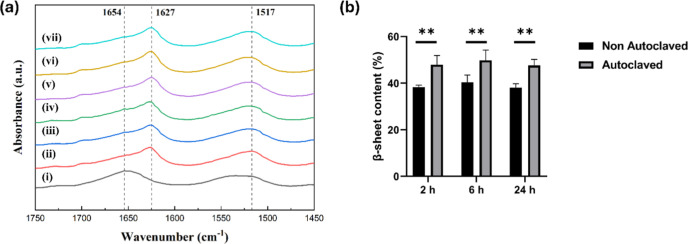
(a) FTIR spectra of samples under the following conditions: (i) untreated, (ii–iv) ethanol treatment for 2, 6, and 24 h, respectively, (v–vii) ethanol treatment for 2, 6, and 24 h, followed by autoclave sterilization (20 min at 121 °C), respectively. (b) Quantification of β-sheet content of nonautoclaved and autoclaved samples. Data are means ± SD (*n* = 3). Statistical significance was determined by one-way ANOVA (**p < 0.01).

### 3.2 FTIR Analysis

Ethanol cross-linking induced a structural transformation in SF, leading to a shift in characteristic spectral peaks. A shift in the amide I region (from 1654 to 1627 cm^–1^) was detected, indicating an increased presence of β-sheet structures and a transition toward a more ordered molecular conformation. This shift is associated with the transition from a random coil structure to a β-sheet structure and has been correlated with reduced water solubility (i.e., cross-linking).^58,59^ Analogously, a shift in amide II (from 1540 to 1517 cm^–1^) region was observed corresponding to N–H bending vibrations. After sterilization by autoclaving, the same peaks were detected (Figure 2), but the β-sheet content increased significantly (reaching ∼48%) in comparison to the nonautoclaved countperparts (∼39%).

### 3.3 Mechanical Properties of Pure SF Hollow Nanoyarns

Stress–strain curves showed higher strain and stress at break for the 10-cycle nanoyarns compared to the 5-cycle counterparts (Figure 3a). The 10-cycle nanoyarn reached 166.4 ± 21.05% strain at break, whereas the 5-cycle nanoyarn showed lower but still remarkable elongation (86.77 ± 12.03%) (Figure 3b). The 10-cycle nanoyarns achieved an UTS of 1.47 ± 0.12 MPa, which was higher than the 5-cycle counterparts (0.90 ± 0.16 MPa) (Figure 3b). This trend was also evident at different ethanol cross-linking times: an increase in the number of spinning cycles increased the UTS (Figures S2 and S3). We observed no significant change in Young’s modulus between 5-cycle and 10-cycle nanoyarns. All tensile testing values are listed in Table S2.

**Figure 3:**
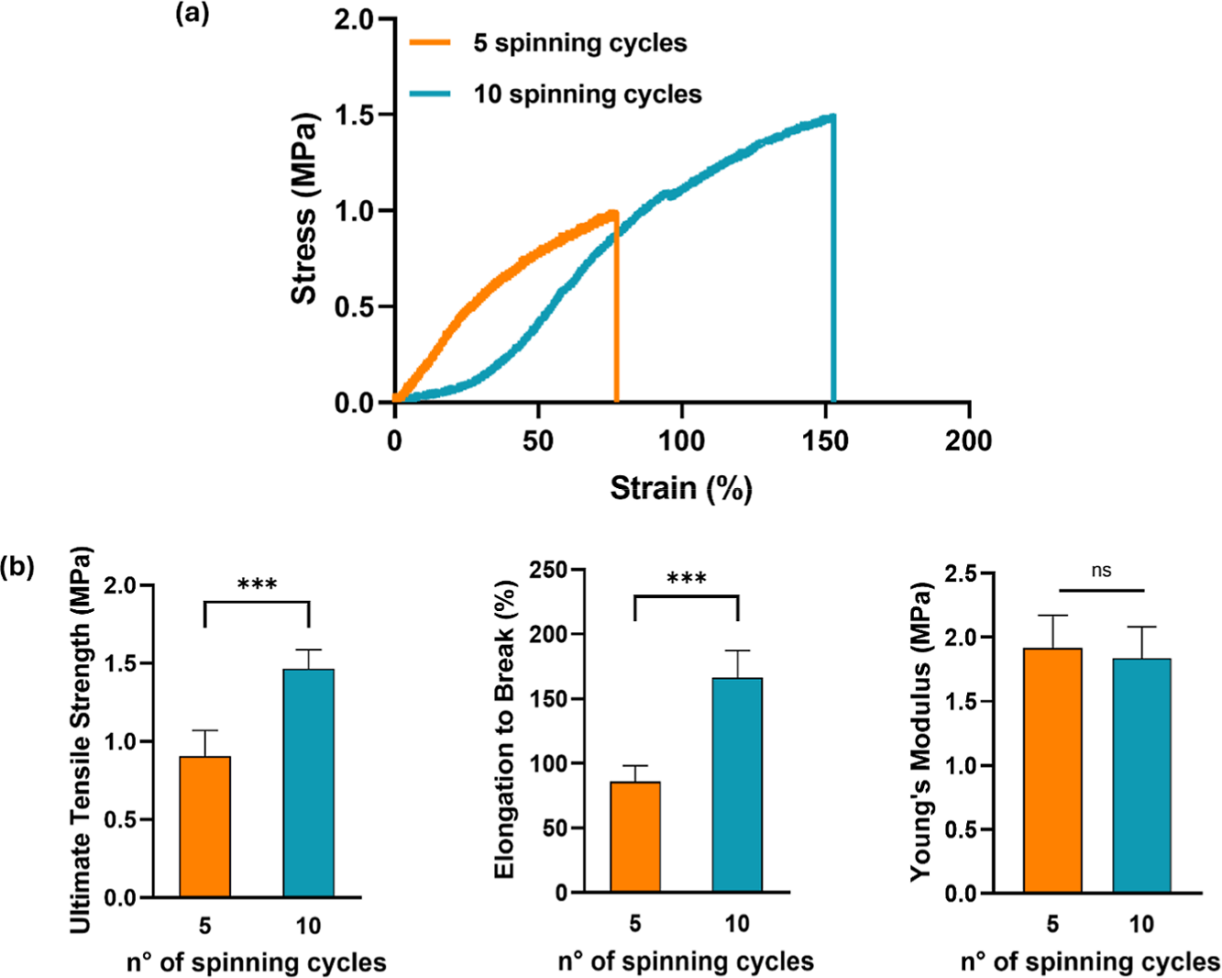
Mechanical characterization of SF nanoyarns. (a) Representative stress–strain curves of the 5-cycle and 10-cycle SF nanoyarns cross-linked with ethanol for 2 h. (b) Comparison of (left to right) ultimate tensile strength, elongation to break, and Young’s modulus for both groups. Data are means ± SD (*n* = 5). Statistical significance was determined using Welch’s *t*-test (***p < 0.001; ns = nonsignificant).

### 3.4 Impact of Autoclaving on the Mechanical Properties of SF Nanoyarns

Next, we measured the tensile properties of SF nanoyarns after terminal sterilization (i.e., autoclaving at 121 °C for 20 min). Figure 4a,b show the representative stress–strain curves of the nonautoclaved and autoclaved nanoyarns following ethanol cross-linking for 2 h.

**Figure 4:**
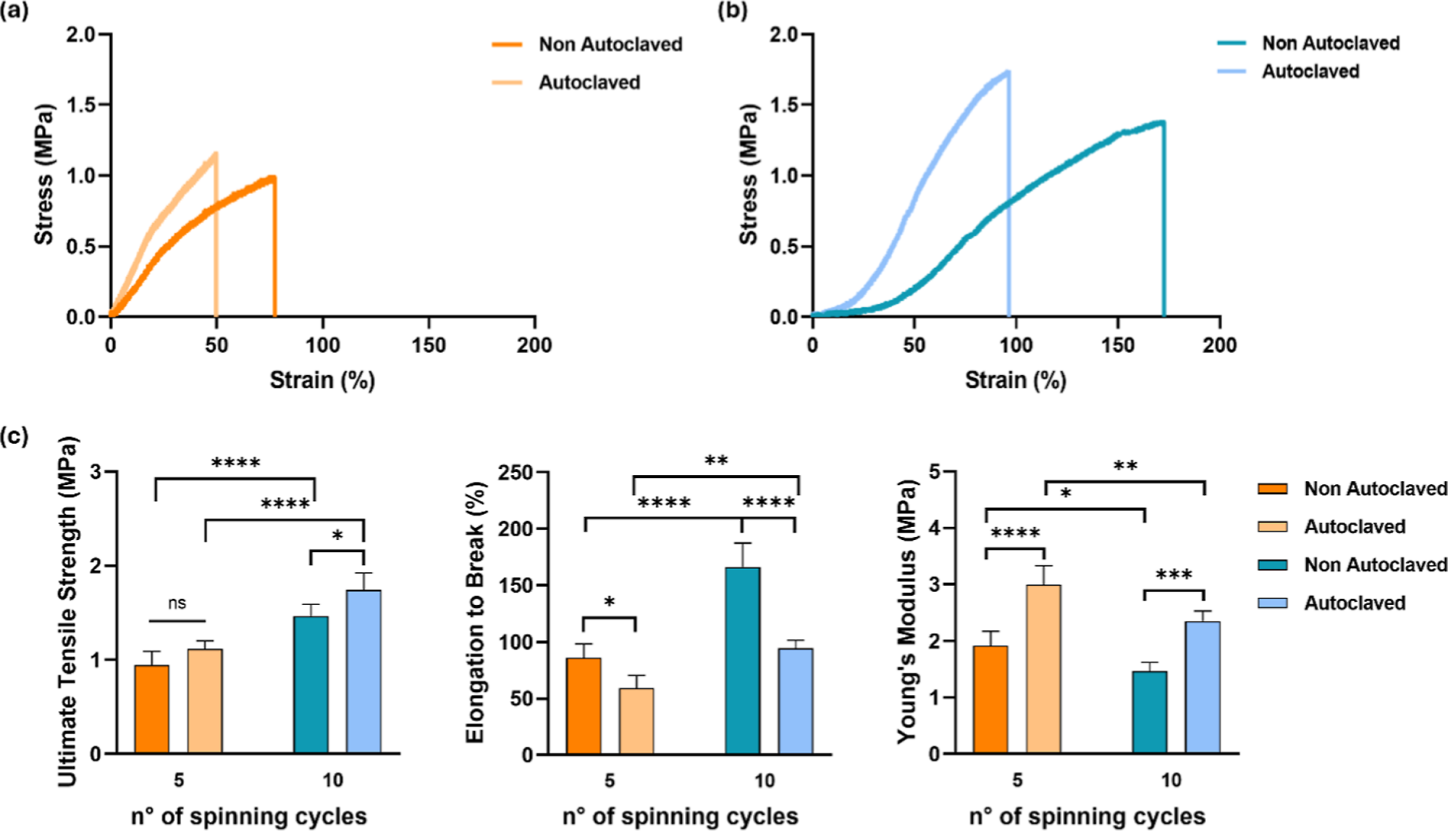
Impact of sterilization on the mechanical properties of SF nanoyarns cross-linked with ethanol for 2 h. (a,b) Representative stress–strain curves of (a) 5-cycle and (b) 10-cycle nanoyarns after autoclaving. (c) Comparison of (left to right) ultimate tensile strength, elongation to break, and Young’s modulus of nonautoclaved and autoclaved samples. Data are means ± SD (*n* = 5). Statistical significance was determined by one-way ANOVA (*p < 0.05, **p < 0.01, ***p < 0.001, ****p < 0.0001).

Autoclaving increased the UTS in 5-cycle nanoyarns and reached statistical significance in 10-cycle nanoyarns (Figures 4). The UTS increased from 0.95 ± 0.144 to 1.12 ± 0.09 MPa for the thinner nanoyarns, and from 1.47 ± 0.12 to 1.75 ± 0.18 MPa for the thicker ones, but both showed a remarkable reduction in the elongation to break upon autoclaving (30% and 43% for the 5-cycle and 10-cycle nanoyarns, respectively), and an increase in Young’s modulus (3.00 ± 0.33 and 2.35 ± 0.18 MPa, respectively). The increase in the Young’s modulus upon autoclaving was paralleled by an increase in the β-sheet content (Figure 2). Stress–strain curves and additional mechanical properties of nonautoclaved and autoclaved nanoyarns following ethanol crosslinking at different time points are presented in Table S3 and Figures S5 and S6.

### 3.5 Cell Viability

The metabolic activity of cells cultured in scaffold-extracted media was comparable to or greater than that of the negative control (Figure 5a–d). The viability of HUVECs and HUASMCs reached 94.55 ± 5.57% and 139.7 ± 5.57%, respectively, after 3 days (Figure 5b–d), confirming the cytocompatibility of the electrospun SF scaffolds. For the two positive controls (50% DMSO and latex), near-zero metabolic activity was detected. Cytocompatibility was supported by fluorescence microscopy (Figure 5e,f), which revealed a uniformly spread cell morphology, consistent with the appearance of both HUVECs and HUASMCs cultured in fresh media (negative control).

**Figure 5:**
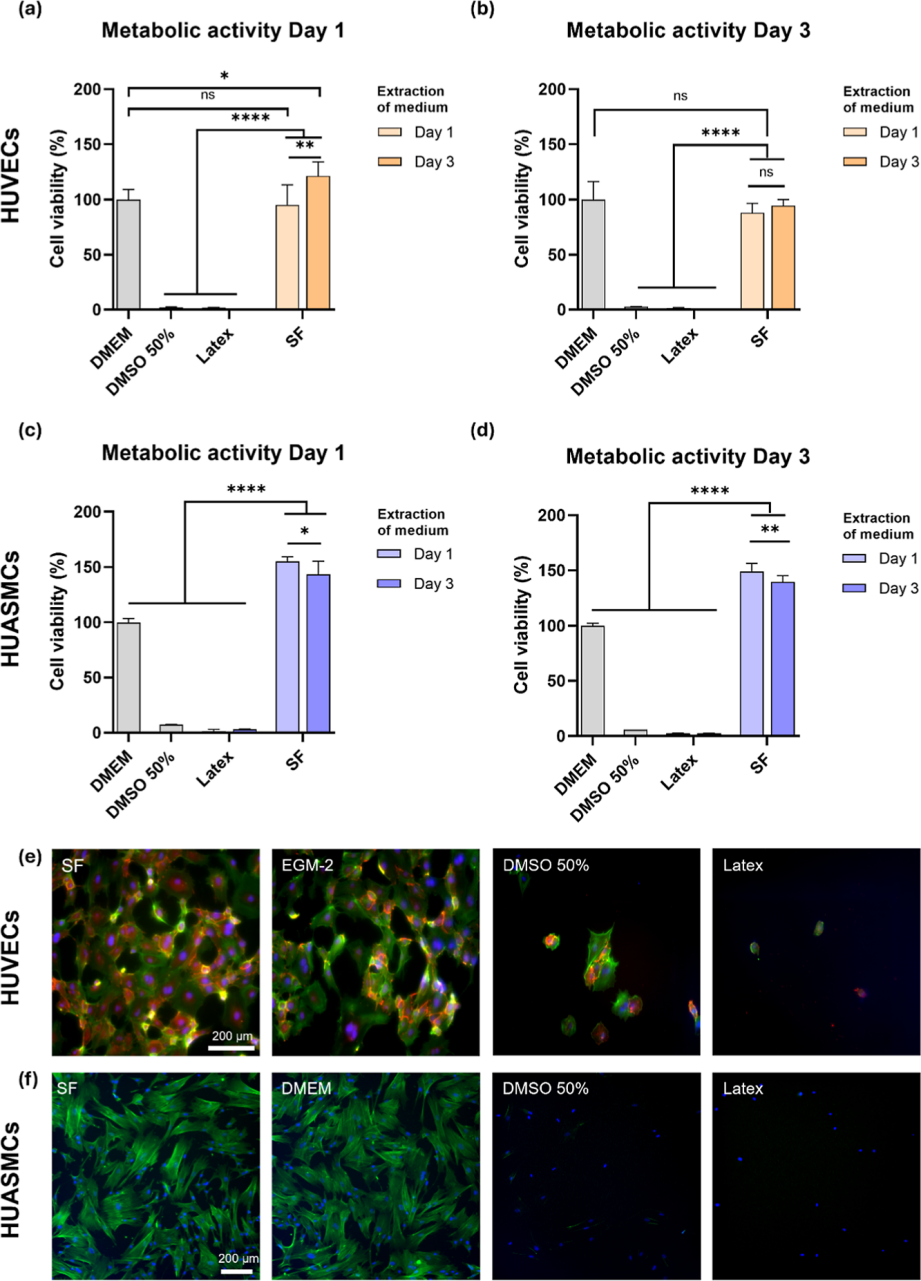
Cytocompatibility assessment. (a,b) Metabolic activity of HUVECs incubated with EGM-2, 50% DMSO, latex, and SF extracted media for (a) 1 day and (b) 3 days. (c, d) Metabolic activity of HUASMCs incubated with DMEM, 50% DMSO, latex, and SF extracted media for (c) 1 day and (d) 3 days. Data are means ± SD (*n* = 5). Statistical significance was determined by one-way ANOVA (*p < 0.05, **p < 0.01, ***p < 0.001, ****p < 0.0001; ns = nonsignificant). (e) Fluorescence microscopy images (day 1 of culture) of HUVECs cultured with EGM-2, 50% DMSO, and extracted media from latex and electrospun SF. Endothelial cells are stained with an anti-CD31 antibody (red). (f) Fluorescence microscopy images (day 1 of culture) of HUASMCs cultured with DMEM, 50% DMSO, and extracted media from latex and electrospun SF. Images in (e,f) are counterstained for actin filaments (phallodin-iFluor488, green) and nuclei (DAPI, blue).

### 3.6 Release of TNF-α by U937 Cells Cultured on SF Scaffolds

The release of TNF-α from U937 cells seeded on SF yarns and mats, as well as on fibrin gels and TCPS, was evaluated after 1 and 3 days in culture. After 1 day, TNF-α levels released by the cells seeded onto the nanoyarns, mats, and fibrin gels were comparable to the value obtained for the M0 (naïve) and M2 (anti-inflammatory, TNF-α negative control), and significantly lower than the TNF-α value obtained from M1 (pro-inflammatory, TNF-α positive control) on TCPS (Figure 6). These results indicate that SF, both in the shape of yarns or mats, did not trigger the release of TNF-α from the seeded U937 cells, suggesting its low inflammatory properties. After 3 days, SF nanoyarns and mats showed TNF-α values comparable to M0 macrophages on TCP. As for day 1, SF did not trigger the release of TNF-α, and values for both nanoyarns and mats were significantly lower than M1 macrophages on TCP, however, significantly higher than for M2 macrophages on TCPS (Figure 6). At both time points, TNF-α values associated with SF scaffolds were similar to those of fibrin, which is a known low-immunogenic material that does not substantially trigger an inflammatory response in vitro and in vivo.^60,61^

**Figure 6:**
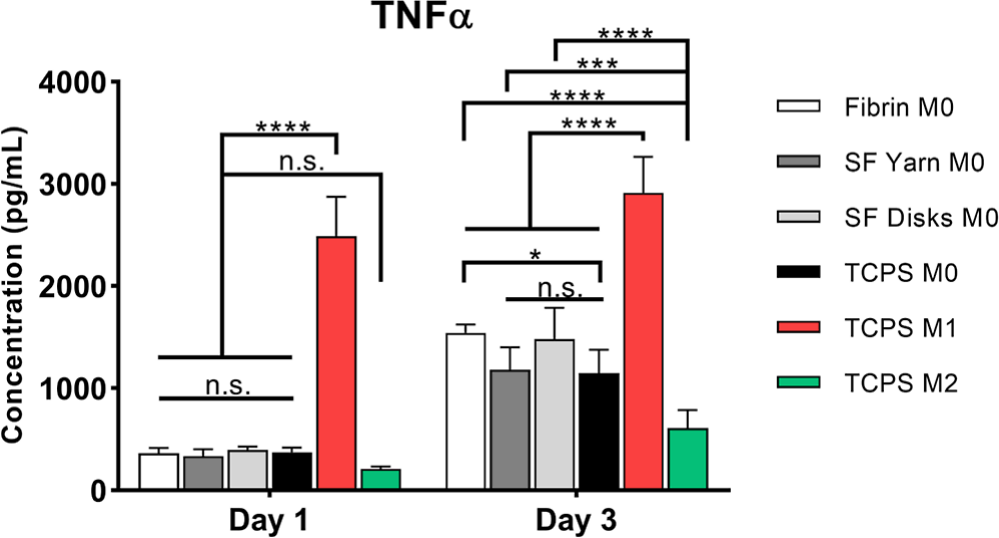
Quantification of TNF-α from U937 cells on fibrin gels, SF yarns, SF mats, and TCPS. For fibrin gels, SF nanoyarns, and SF mats (disk shape), U937 were supplemented only with PMA to maintain their naïve (M0) polarization state. For TCPS, in addition to M0 macrophages, M1 (pro-inflammatory; 100 ng/mL LPS and 20 ng/mL IFN-γ) and M2 (anti-inflammatory; 20 ng/mL IL-4 and IL-13). The quantification was performed after 1 and 3 days of culture. Data are means ± SD (*n* = 5). Statistical significance was determined by one-way ANOVA (*p < 0.05, **p < 0.01, ***p < 0.001, ****p < 0.0001; ns = nonsignificant).

### 3.7 Feasibility of the Bottom-Up Approach

The use of fibers in bottom-up technologies, such as those inspired by textile principles, enables the engineering of intricately structured tissues and organs. We tested the feasibility of using our developed SF-nanoyarns to create hierarchically organized structures by knotting, twisting, and weaving (Figure 7). The knot (Figure 7a) maintained its configuration after handling, drying, and imaging, demonstrating adequate structural integrity. The woven meshes (Figure 7d,e) exhibited a uniform weave pattern with consistent nanoyarn alignment and were intentionally fabricated with and without pores, following distinct textile patterns. Fiber integrity was preserved in the knots and meshes, confirming the successful fabrication of reproducible and structurally coherent textile assemblies.

**Figure 7:**
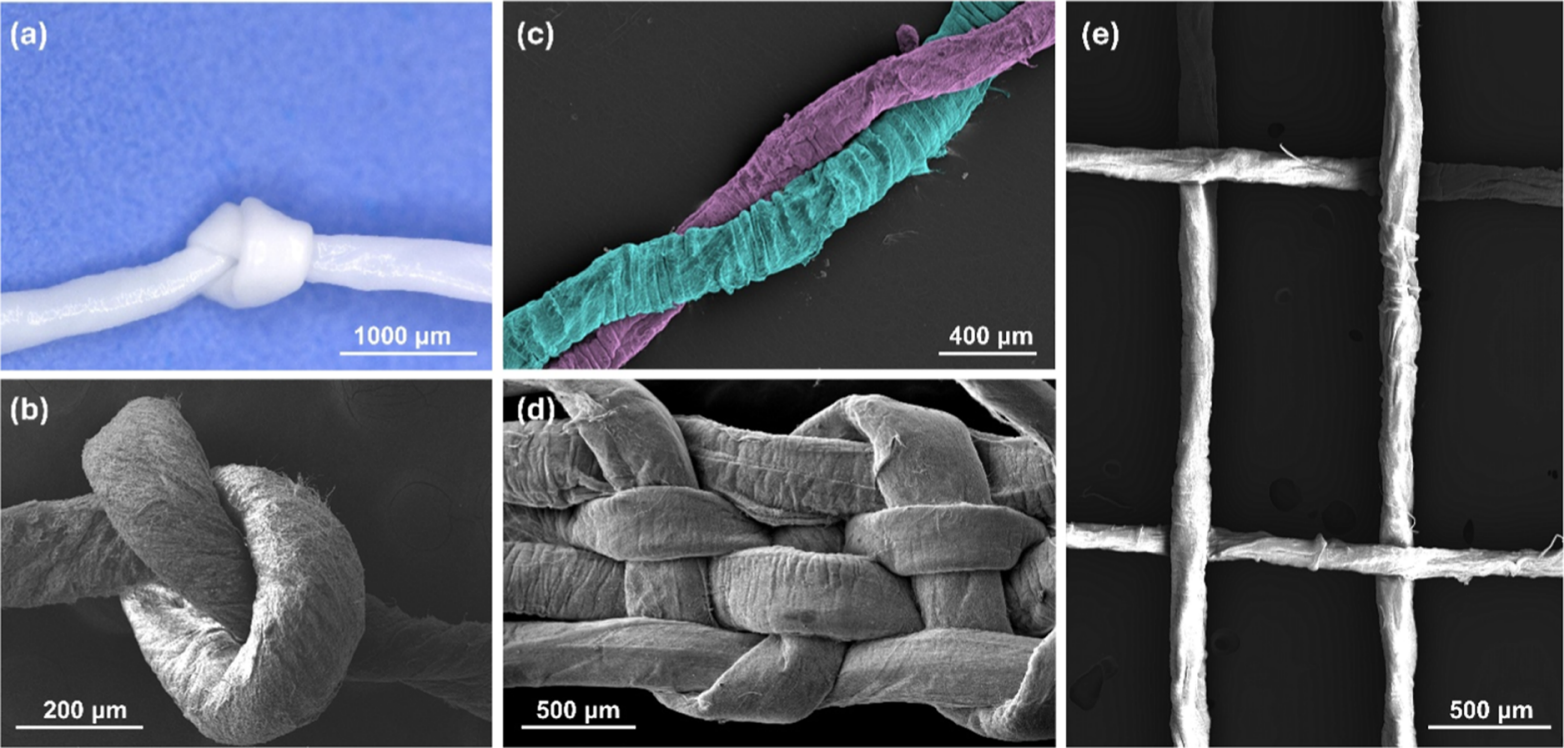
Feasibility of the bottom-up approach. (a) Brightfield image of knotting. (b) SEM image of a knot formed by SF nanoyarns. (c) SEM image of twisted SF nanoyarns, colored using MountainsSEM to facilitate visualization. (d,e) SEM images of meshes woven from SF nanoyarns, showcasing different porosities.

## 4 Discussion

This study explored the fabrication of pure SF nanoyarns and evaluated the impact of stabilization treatments on their mechanical properties, as well as their potential assembly into textile-based configurations. We successfully prepared long SF nanoyarns, with adequate tensile strength and elasticity, which were compatible with autoclaving and did not inhibit the metabolic activity of HUVECs or HUASMCs. Their flexibility also enabled bottom-up assembly into textile constructs by weaving, highlighting their potential as biobased building blocks for medical textiles.

Only a few studies have investigated the processing of SF into nanoyarns by funnel electrospinning, and in all previous cases, this required a combination of SF with natural proteins or synthetic polymers, which were an integral part of the final yarn.^46,52^ In contrast, we focused on the production of nanoyarns composed of pure SF, which also featured a hollow shape to enhance structural complexity. Fabricated hollow nanoyarns were produced by integrating a guiding thread into a custom-made funnel electrospinning setup. This was achieved after systematically optimizing the process parameters to yield a continuous form. Key operating parameters included the funnel and winder speed and flow rate because these underpin the formation and stability of the fibrous cone.^14,23,24^ Specifically, we achieved a stable process with a funnel rotation speed of 450 rpm and an overall flow rate of 1 mL/h. Higher rotation speeds and flow rates triggered the abrupt disappearance of the fibrous cone or led to uneven nanoyarn thickness, respectively. Thus, in our fabrication, we used a relatively low rotation speed compared to the values reported in studies where SF is blended with other polymers.^46,52^ As an alternative, adding one or more syringes could further enhance nanofiber deposition at the funnel’s periphery, thereby resulting in a more uniform nanoyarn wall thickness.^62,63^ However, the feasibility of this modification depends on the spatial constraints of the experimental setup.

A tensioner was needed for the guiding thread, thereby facilitating continuous collection from the winder system. Two different types of nanoyarns were developed, categorized according to the number of spinning cycles. The resulting core–sheath structures were composed of an inner guiding PVA thread and an outer nanofibrillar SF sheath. The stabilization of the sheath by ethanol treatment, followed by the removal of the PVA yarn by immersion in water, resulted in pure hollow SF nanoyarns, the first of their kind.

The average outer diameter of the nanoyarns was easily adjusted by changing the number of spinning cycles. Five cycles produced an outer diameter of 503.80 ± 27.14 μm, whereas 10 cycles increased this to 593 ± 47.04 μm (Table S1). Regenerated SF materials often contain a substantial number of amorphous structures such as random coils, α-helices, side chains, turns, and bends.^64^ These noncrystalline configurations are loosely organized, and their weak intermolecular forces contribute to the material’s solubility in water, thus compromising its mechanical strength. To address these limitations, postfabrication treatments are used to increase the formation and stability of ordered crystalline structures (β-sheets), substantially modifying the scaffold’s stability and mechanical resistance. We focused on promoting the crystallization of SF nanoyarns by ethanol treatment for different time periods. FTIR results (Figure 2) revealed a peak shift in the amide I region, typically attributed to the formation of β-sheets^58^ indicating conformational transition to a water-insoluble state. Ethanol acts as a dehydrating agent, lowering the dielectric constant of the medium and removing bound water from SF chains, which favors inter- and intramolecular hydrogen bonding, thereby stabilizing β-sheet crystalline domains at the expense of random-coil and α-helical conformations.^58^ This treatment enabled the precise preservation of the morphology of the nanofibers composing the SF nanoyarns, even when exposed to an aqueous environment (Figure S6c).

The analysis of stress–strain curves (Figure 3) during tensile tests showed changes in UTS and elongation to break between the nanoyarns with different spinning cycles, which were mainly attributed to their initial morphology. The UTS of the thicker nanoyarns was significantly higher compared to the thinner ones, reflecting the denser structure. For example, in the 2 h ethanol treatment group, the UTS was 1.47 ± 0.12 MPa and 0.90 ± 0.16 MPa for thicker and thinner nanoyarns, respectively (Figure 3b). In parallel, the stress–strain curves revealed a significantly higher elongation to break for 10-cycle nanoyarns (166.40 ± 21.05%) than the 5-cycle analogs (86.77 ± 12.03%) (Figure 3b). This analysis of mechanical behavior offers important insights into how the thickness of the nanoyarn affects its overall performance. These data support the design and optimization of SF nanoyarns produced by funnel electrospinning, enabling greater control over their mechanical properties and expanding the potential for application-specific customization. One of the essential prerequisites for the translation of biomaterials is sterilization. Steam sterilization (or autoclaving) is a widely used method based on exposure to high-pressure saturated steam at temperatures of 121 °C for at least 15 min.^65^ Autoclaving is frequently applied due to its proven efficacy and operational simplicity, and it is considered the preferred terminal sterilization method by the European Medicines Agency. One particular advantage of SF compared to other protein-based materials is that steam sterilization has no detrimental effects. Indeed, autoclaving significantly increases its β-sheet content.^36,66^ We thoroughly investigated the impact of steam sterilization on the mechanical properties of our SF nanoyarns. Stress–strain analysis indicated that the autoclaved samples tended to show the highest UTS and Young’s modulus, with a concomitant decrease in elongation to break (Figures 4, S4 and S5). These results agree with previous studies showing that steam sterilization not only ensures sample sterility but also contributes to further stabilization of the scaffold.^42,55,66^ Importantly, the fiber morphology remained intact upon sterilization (Figure S6).

FTIR analysis (Figure 2) confirmed that all samples (with and without autoclaving) exhibited a characteristic peak at 1617–1627 cm^–1^ and 1696–1700 cm^–1^, indicating β-sheet formation, as previously reported.^58,66,67^ The β-sheet content increased significantly in the autoclaved samples (reaching ∼48%) in comparison to the samples treated only with ethanol (∼39%), as previously reported.^68^ Accordingly, the autoclaved samples exhibited similar or higher performance in terms of UTS, thus indicating that steam sterilization did not disrupt the β-sheet network but rather refines its organization. These findings support the hypothesis that steam sterilization promotes thermodynamically driven reorganization of crystalline β-sheet domains into larger and more compact aggregates,^69^ thus enhancing structural cohesion and mechanical integrity without compromising the characteristic β-sheet structures of SF. In addition, Gil and colleagues have demonstrated that autoclaving processes also impact the organization of the amorphous regions. Specifically, steam sterilization (high heat, pressure, and moisture) disrupts hydrogen bonds and hydrophobic interactions in the amorphous regions, promoting noncovalent realignments like tighter chain packing or partial ordering.^70^ Overall, the exposure of SF to steam sterilization provides changes in (i) β-sheet content,^59^ (ii) crystalline domain size, and (iii) amorphous-phase supramolecular organization,^70^ which can explain the stiffness and embrittlement of the SF nanoyarns. These changes contributed to provide long-term mechanical stability.^66^ Such stability was also reflected in the low degradation profile of our SF nanoyarns (<2%) after 2 weeks in water (Figure S7).

Cytocompatibility assessment is required to ensure the safety and clinical viability of new scaffold materials. HUVECs and HUASMCs were used to determine the effect of the electrospun SF material on cell survival according to ISO 10993-5. Those tests showed that SF supported metabolic activity approaching or exceeding 100%, confirming excellent cytocompatibility (Figure 5a–d). These results agree with previous studies reporting the high biocompatibility of SF-based materials.^71,72^ Our quantitative findings were further corroborated by fluorescence microscopy (Figures 5e,f, and S8 and S9). The expression of CD31 in HUVECs indicates the preservation of their endothelial phenotype. Furthermore, HUASMCs showed clearly defined nuclei as well as spread and well-defined actin filaments, indicating normal cytoskeletal organization.

Additional biological characterization showed that TNF-α secretion from macrophage-like cells cultured on SF scaffolds (Figures 6 and S10) was similar to M0 controls and lower than M1 macrophages. SF, in both nanoyarn and mat forms, did not induce a pro-inflammatory response. TNF-α levels on SF matched those on fibrin, confirming its low immunogenicity. Further characterization by endotoxin quantification showed levels of endotoxins well below the limit of 0.5 EU/mL stated in the ISO-11737:3 (Figure S11). Additionally, the SF nanoyarns showed a swelling ratio of 203.7 ± 25.46% (Figure S12), which corresponds to hydrophilic properties. This high value is consistent with a highly porous polymer network capable of extensive hydration (Video S1), which is essential for replicating the natural moist environment of tissues in biomedical applications.

Textiles such as woven meshes and knot-based assemblies are ideal as scaffolds for tissue engineering applications.^73,74^ The mechanical flexibility and structural integrity demonstrated by the nanoyarns enabled their bottom-up assembly into textile prototypes (Figure 7). Textile-based approaches allow the production of highly organized, anisotropic architectures with tunable porosity, allowing them to mimic the hierarchical structure of native tissues.^16^ In particular, the remarkable capacity of the nanoyarns to form knots without breaking could also be useful in techniques like knitting. The formation of 3D geometry is especially beneficial for tissue engineering and is currently used in the medical implant industry.^75^ Textile manufacturing techniques have been integrated with tissue engineering to develop structurally and functionally woven meshes or tubular scaffolds. For example, a blend of tropoelastin and SF has been electrospun into nanofibrous yarns and subsequently assembled into woven meshes.^52^ The cultivation of fibroblasts demonstrated the suitability of these meshes for applications such as pelvic organ prolapse repair. In addition, a woven tissue-engineered vascular graft (TEVG) was developed using cell-assembled extracellular matrix (CAM) yarns produced entirely by human fibroblasts.^76^ More recently, elastin-like recombinamers were processed into meter-long fibers, which exhibited remarkable elasticity and mechanical robustness, facilitating the adhesion and proliferation of HUVECs.^77^ These fibers were assembled into highly ordered structures by braiding, weaving and knitting, confirming the potential to fabricate complex scaffolds suitable for vascular prosthesis. Fiber technologies that rely on biobased resources could advance future innovations in medical textiles and tissue engineering by providing mechanically robust hierarchical structures that support proper cell function, proliferation and differentiation.

## 5 Conclusion

This study has presented the first pipeline for the reproducible production of pure SF hollow nanoyarns. Our systematic investigation determined the influence of ethanol treatment and autoclaving on the performance of the nanoyarns, demonstrating their compatibility with terminal sterilization. This allowed us to elucidate the role of the postprocessing steps on the mechanical stability and elasticity of the SF. All scaffolds showed high cytocompatibility, supporting the use of these materials for tissue engineering. We also demonstrated that the nanoyarns were compatible with the assembly of textile prototypes (i.e., woven meshes), which underpins their potential in the field of medical textiles.

## Supplementary Material





## Data Availability

The data that support the findings of this study are available from the corresponding author upon reasonable request.

## Notes

Quantification of wall-thickness and diameter of SF nanoyarns; mechanical characterization of SF nanoyarns following ethanol treatment at different time points; impact of autoclaving on their mechanical properties; evaluation of nanoyarn surface and fiber morphology before and after autoclaving; degradation analysis of SF nanoyarns in aqueous environment; fluorescence microscopy images of HUVECs and HUASMCs upon indirect cytotoxicity testing; confocal microscopy images of U937 cells cultured on electrospun SF mats; determination of endotoxin levels; swelling ratio (PDF)
Video S1: Hydrophilic properties of electrospun SF disks (MP4)
