# Supplementary material for: Manufacturing Silk Fibroin Hollow Nanoyarns as Fundamental Units for Advanced Medical Textiles
Source: ACS Appl Mater Interfaces. 2026 Mar 17;18(12):17457–69. doi: 10.1021/acsami.5c23471 (PMC13051439; doi:10.1021/acsami.5c23471)
Supplement: Supplementary file 1 [file am5c23471_si_001.pdf]

Supporting Information

Manufacturing silk fibroin hollow nanoyarns as fundamental units for advanced medical textiles

Athanasios Papakonstantinou[1], Maria Gabriella Fois[1], Sergio Acosta[1], Stephan Rütten[2], Alexander Kopp[3], Stefan Jockenhoevel[1], Alicia Fernández-Colino[1]*

[1]Department of Biohybrid & Medical Textiles (BioTex) AME—Institute of Applied Medical Engineering Helmholtz Institute, RWTH Aachen University, Aachen, 52074, Germany

[2]Electron Microscopy Facility Uniklinik RWTH Aachen University, Aachen, 52074, Germany

[3]Fibrothelium GmbH, Aachen, 52068, Germany

*E-mail: fernandez@ame.rwth-aachen.de

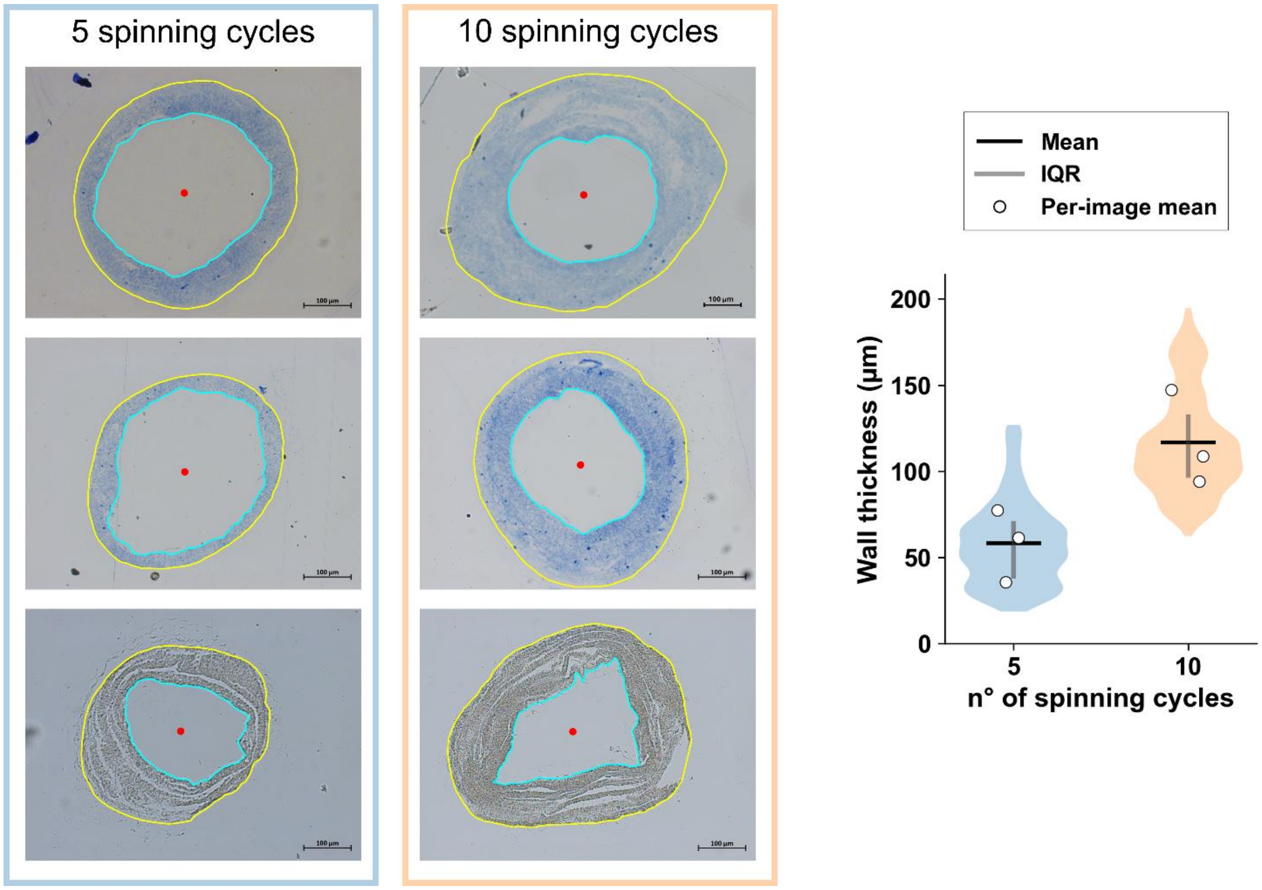

Figure S1. Brightfield microscopy cross-sections of hollow nanoyarns fabricated by 5- and 10- spinning cycles, and analysis of their wall-thickness. The thickness of hollow nanoyarns was quantified by manual tracing of inner and outer contours (blue and yellow lines) in ImageJ. For each image, both contours were analysed together, and their combined centroid (red dot) was used to define the geometric center. Radial wall thickness was calculated automatically as the difference between the outer and inner contour radii at 0.5° angular intervals. The mean wall thickness values for each nanoyarn are represented with a white dot. Thickness distributions are represented for each experimental category (i.e., 5 spinning cycles vs 10 spinning cycles, *n=3*).

Table S1. Outer diameter of SF nanoyarns prior tensile testing. Data are means ± SD (*n = 30*).

	**5 spinning cycles**	**10 spinning cycles**
**Diameter (µm)**	503.80 ± 27.14	593 ± 47.04

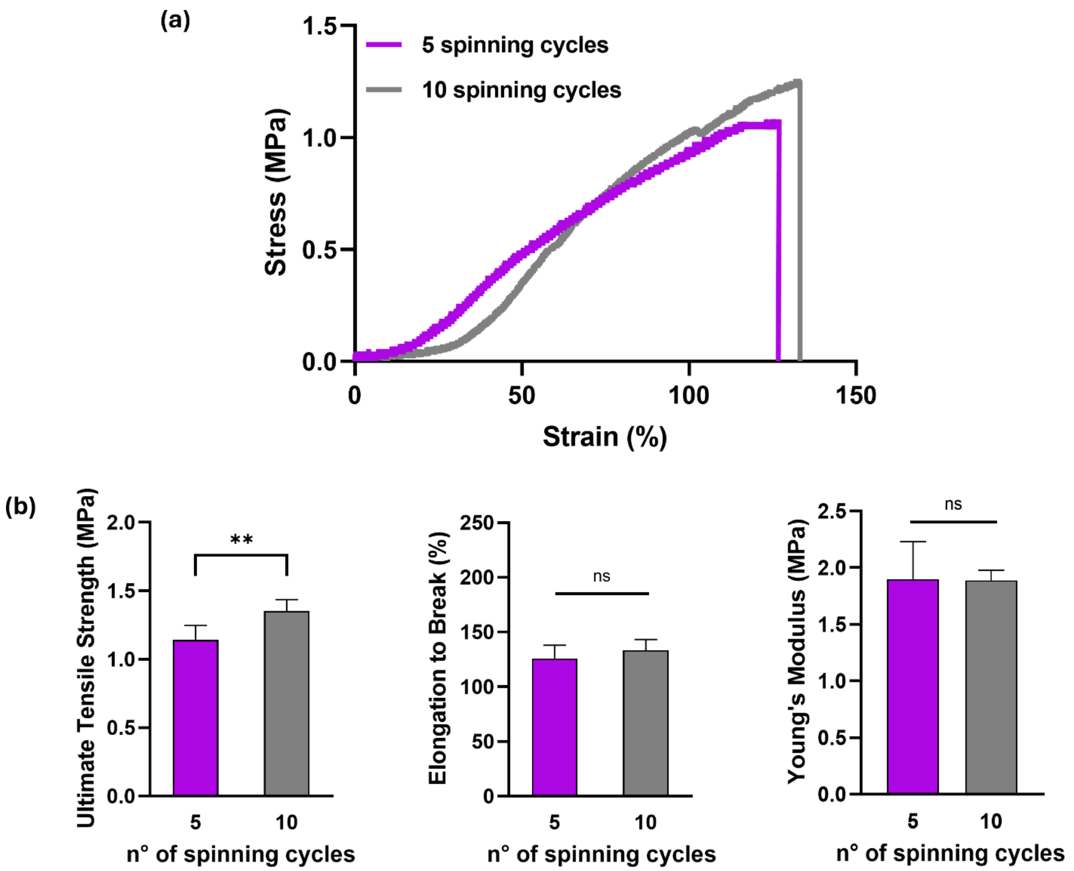

Figure S2. Mechanical characterization of SF nanoyarns. (**a**) Representative stress-strain curves of the 5-cycle and 10-cycle SF nanoyarns crosslinked with ethanol for 6 h. (**b**) Comparison of (left to right) ultimate tensile strength, elongation to break, and Young's modulus for both groups of nanoyarns. Data are means ± SD (*n = 5*). Statistical significance was determined using a Welch's t-test (***p < 0.01; ns = non-significant*).

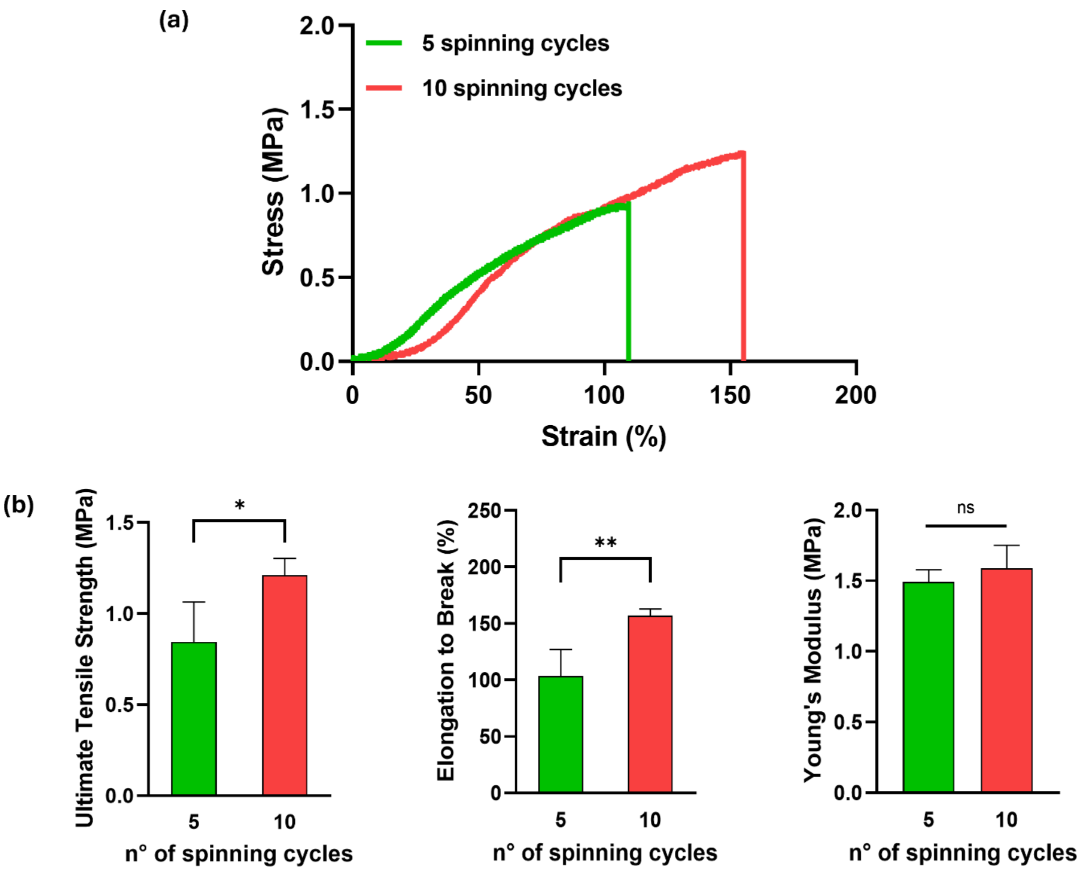

Figure S3. Mechanical characterization of SF nanoyarns. (**a**) Representative stress-strain curves of the 5-cycle and 10-cycle SF nanoyarns crosslinked with ethanol for 24 h. (**b**) Comparison of (left to right) ultimate tensile strength, elongation to break, and Young's modulus for both groups. Data are means ± SD ($n = 5$). Statistical significance was determined using Welch's t-test (*$*p < 0.05$, $**p < 0.01$, and ns = non-significant*).

Table S2. Tensile properties of SF electrospun nanoyarns after ethanol (EtOH) treatment. Data are means ± SD (*n* = 5).

EtOH treatment time	UTS (MPa)		Elongation to Break (%)		Young's Modulus (MPa)	
	5 spinning cycles	10 spinning cycles	5 spinning cycles	10 spinning cycles	5 spinning cycles	10 spinning cycles
2 h	0.95 ± 0.144	1.47 ± 0.12	86.28 ± 12.14	166.40 ± 21.05	1.92 ± 0.26	1.47 ± 0.15
6 h	1.14 ± 0.11	1.35 ± 0.08	126.10 ± 12.13	133.40 ± 9.91	1.90 ± 0.33	1.89 ± 0.09
24 h	0.84 ± 0.22	1.21 ± 0.09	103.40 ± 23.65	157.0 ± 5.82	1.49 ± 0.09	1.69 ± 0.36

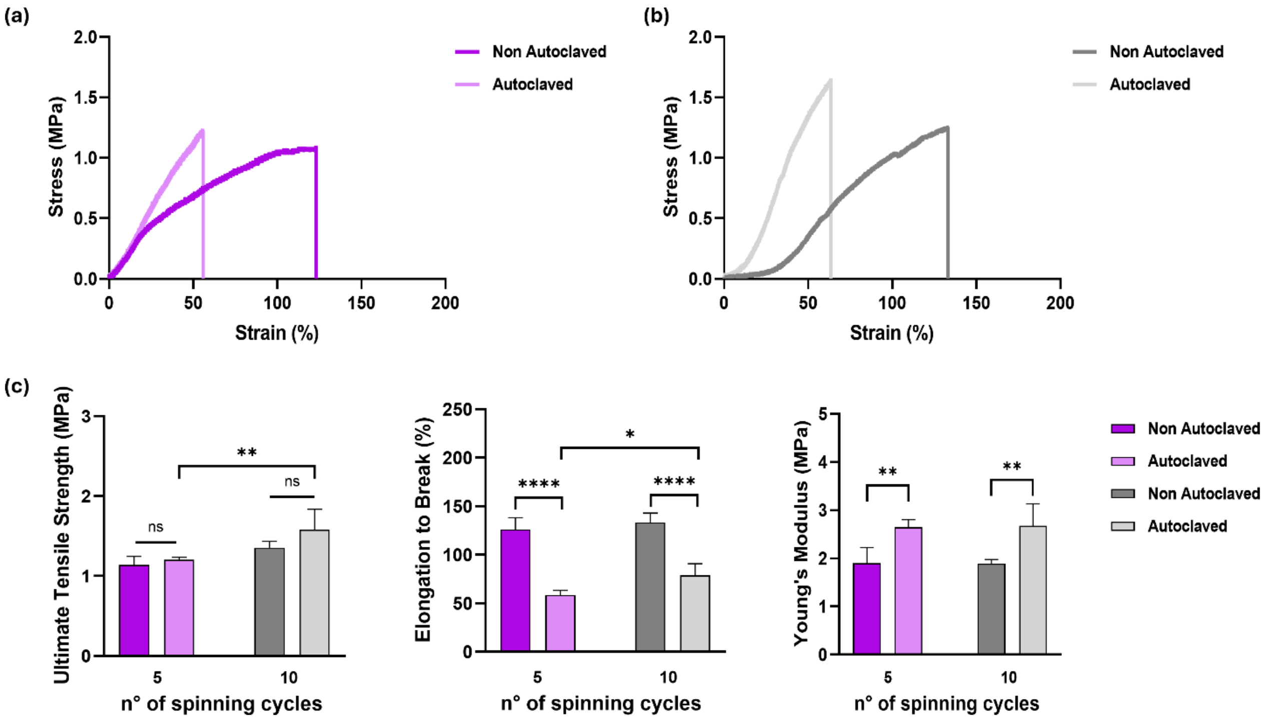

Figure S4. Impact of sterilization on the mechanical properties of the SF nanoyarns crosslinked with ethanol for 6 h. **(a-b)** Representative stress-strain curves of (**a**) 5-cycle and (**b**) 10-cycle nanoyarns after autoclaving. (**c**) Comparison of (left to right) ultimate tensile strength, elongation to break, and Young's modulus of non-autoclaved and autoclaved samples. Data are means ± SD (*n* = 5). Statistical significance was determined by one-way ANOVA *(*p < 0.05, **p < 0.01, ***p < 0.001, ****p < 0.0001, and ns = non-significant)*.

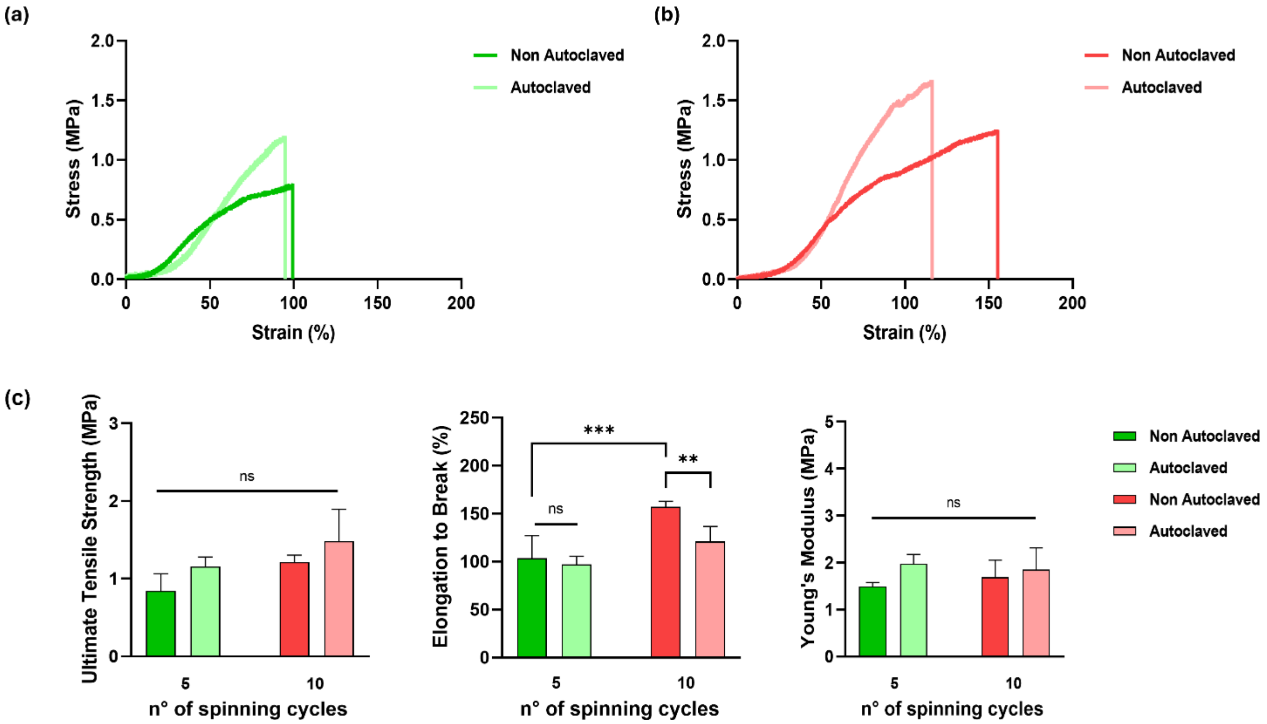

Figure S5. Impact of sterilization on the mechanical properties of the SF nanoyarns crosslinked with ethanol for 24 h. (**a-b)** Representative stress-strain curves of (**a**) 5-cycle and (**b**) 10-cycle nanoyarns after autoclaving. (**c)** Comparison of (left to right) ultimate tensile strength, elongation to break, and Young's modulus of non-autoclaved and autoclaved samples. Data are means ± SD (*n* = 5). Statistical significance was determined by one-way ANOVA *(*p < 0.05, **p < 0.01, ***p < 0.001, ****p < 0.0001, and ns = non-significant*).

Table S3. Tensile properties of SF electrospun nanoyarns after ethanol (EtOH) treatment and sterilization by autoclaving. Data are means ± SD (*n = 5*).

**EtOH treatment time**	**UTS (MPa)**		**Elongation to Break (%)**		**Young's Modulus (MPa)**	
	**5 spinning cycles**	**10 spinning cycles**	**5 spinning cycles**	**10 spinning cycles**	**5 spinning cycles**	**10 spinning cycles**
2 h	1.12 ± 0.09	1.75 ± 0.18	59.50 ± 10.99	94.40± 7.26	3.00 ± 0.33	2.35 ± 0.18
6 h	1.20 ± 0.03	1.58 ± 0.26	58.56 ± 4.86	78.77 ± 12.13	2.65± 0.16	2.68 ± 0.46
24 h	1.16 ± 0.12	1.48 ± 0.41	96.93 ± 8.68	121.0 ± 15.65	1.98 ± 0.20	1.85 ± 0.46

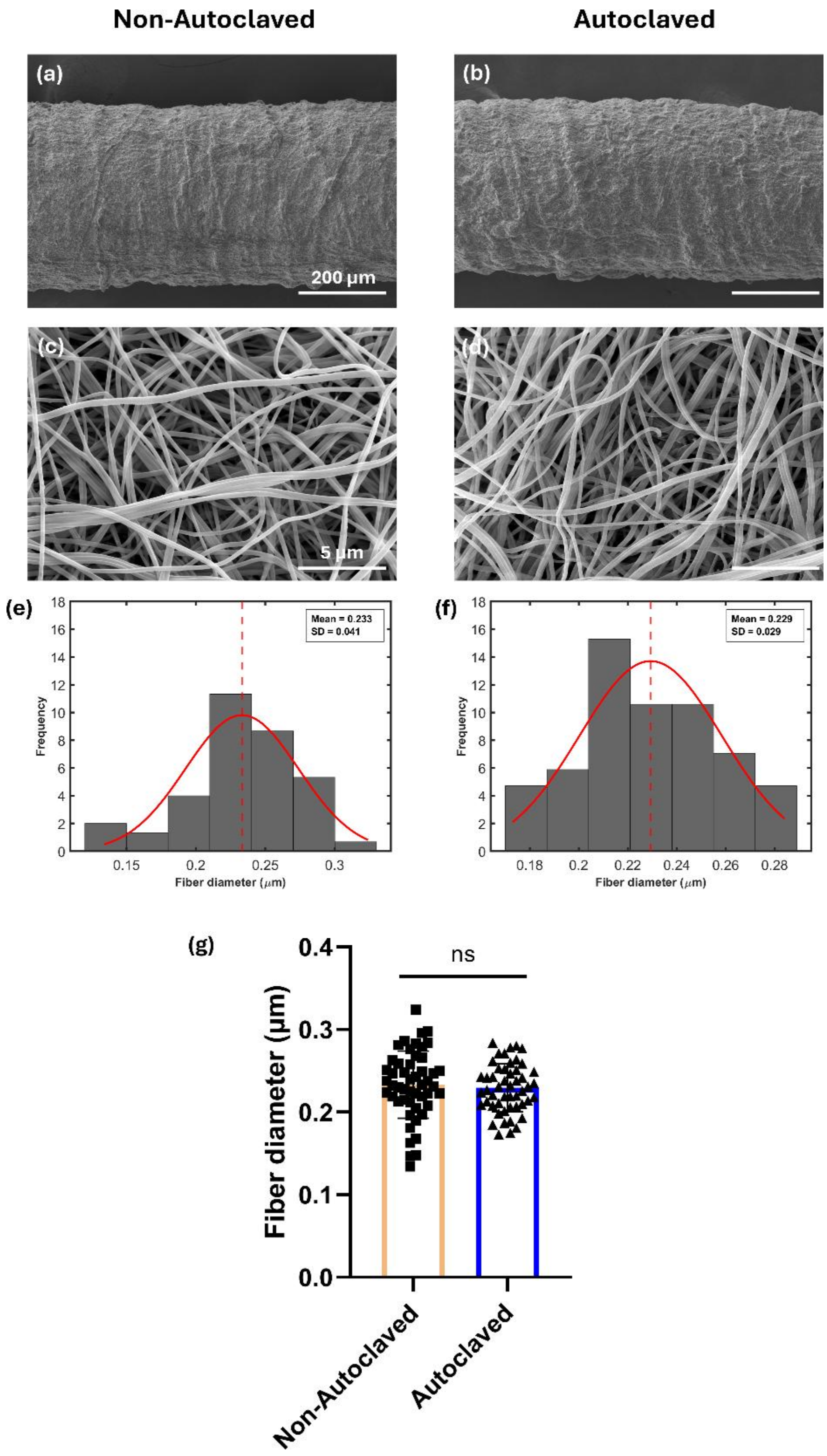

Figure S6. SEM images of nanoyarn surface and fiber morphology: (**a**), (**c**) non-autoclaved, and (**b**), (**d**) autoclaved samples. (**e**), (**f**) Fiber diameter distribution curves. Data are means ± SD (*n = 50* fibers per sample). (**g**) Column chart presenting the average fiber diameter at each condition. Data are means ± SD (*n = 50* fibers per sample).

SF nanoyarns degradation in aqueous environment

The biodegradation test showed that 1.75 ± 0.43% of SF (relative to the initial dry mass) was released into the incubation water after 2 weeks at 37°C.

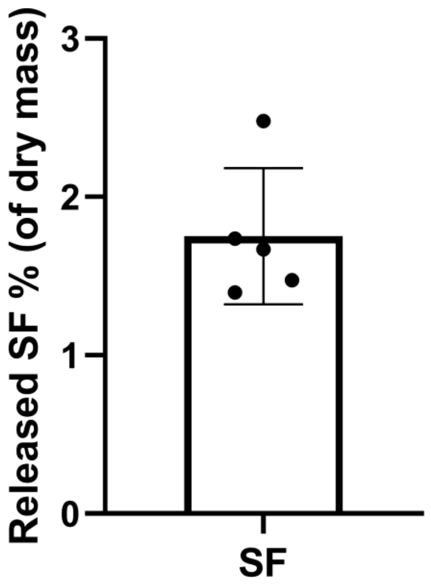

Figure S7. Released % of SF in cell-culture water after 2 weeks of incubation. Data are means ±SD (n=5).

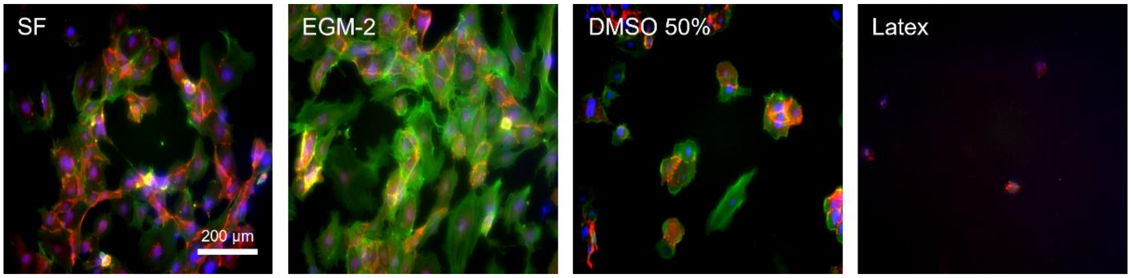

Figure S8. Fluorescence microscopy images (day 3 of culture) of HUVECs with EGM-2, 50% DMSO, and extracted media from latex (positive control) and electrospun SF. Endothelial cells are stained with an anti-CD31 antibody (red). Images are counterstained for actin filaments (phallodin-iFluor488, green) and nuclei (DAPI, blue).

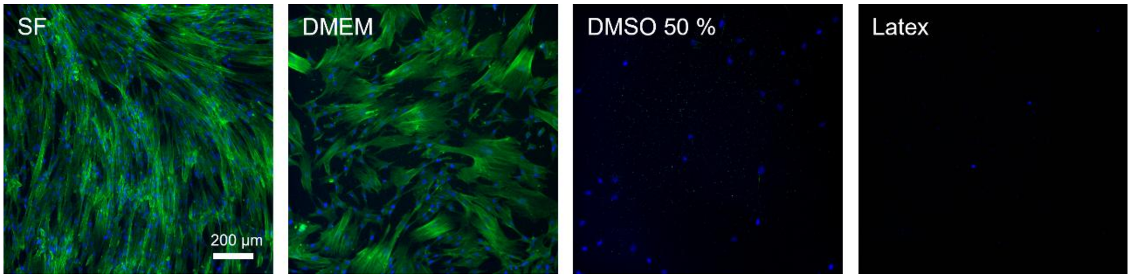

Figure S9. Fluorescence microscopy images (day 3 of culture) of HUASMCs with DMEM, 50% DMSO, and extracted media from latex, and electrospun SF. Images are counterstained for actin filaments (phallodin-iFluor488, green) and nuclei (DAPI, blue).

U937 adhesion on SF scaffolds

The adhesion of U937 cells on SF disks was evaluated through confocal fluorescence microscopy after 1 and 3 days. The macrophages adhered and spread over the course of 1 day (**Figure S10a**). The cells appeared evenly distributed and homogeneous in shape, exhibiting a round morphology typical of monocytic-like cells.[1] After 3 days, proliferation was qualitatively observed, with the cells assembling in tightly packed clusters (**Figure S10b**). Collectively, U937 adhesion and proliferation on the SF scaffolds were confirmed, in support of the use of SF scaffolds as suitable substrate for the cell microenvironment.

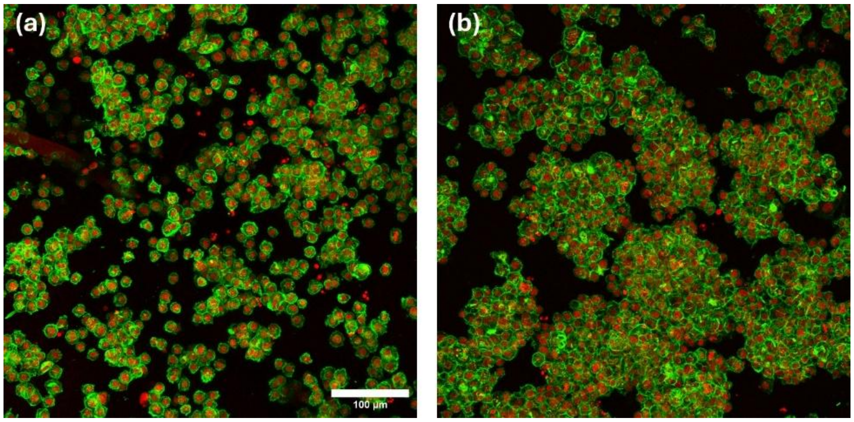

Figure S10. Confocal fluorescence images of U937 cells on SF mats after (a) 1 and (b) 3 days of culture. Cells' cytoskeleton is stained with phalloidin (in green), and cell nuclei are stained with Draq5 (in red). The scale bar represents 100 µm and applies to both subfigures.

Determination of endotoxin levels

The endotoxin detection test showed that the SF nanoyarns have little or no endotoxin. Particularly, SF nanoyarns showed 0.07911 EU/mL, significantly lower than the positive and control, and the maximum allowable level of endotoxin (0.5 EU/mL) according to ISO-11737:3.[2]

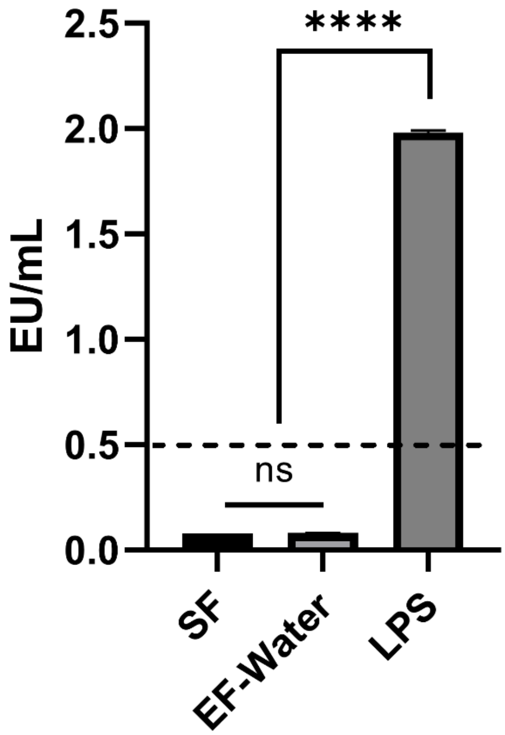

Figure S11. Endotoxin levels of electrospun SF nanoyarns. Data are means ± SD (n=5). Statistical significance was determined by one-way ANOVA (*****p < 0.0001; ns = non-significant*).

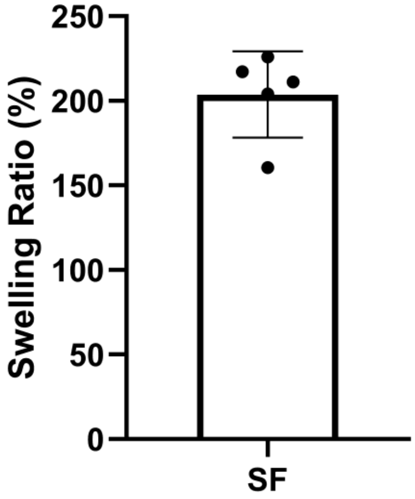

Figure S12. Swelling ratio of electrospun SF nanoyarns. Data are means ± SD (*n* = *5*).

References

(1) Lee, H.; Stachelek, S. J.; Tomczyk, N.; Finley, M. J.; Composto, R. J.; Eckmann, D. M. Correlating Macrophage Morphology and Cytokine Production Resulting from Biomaterial Contact. *J Biomedical Materials Res* **2013**, *101A* (1), 203–212. https://doi.org/10.1002/jbm.a.34309.

(2) ISO 11737-3: Sterilization of Health Care Products - Microbiological Methods Part 3: Bacterial Endotoxin Testing, 2023.
